# On Transducing Properties of Kombucha–Proteinoid Complexes

**DOI:** 10.1021/acsabm.4c00535

**Published:** 2024-06-20

**Authors:** Panagiotis Mougkogiannis, Anna Nikolaidou, Andrew Adamatzky

**Affiliations:** UWE, Unconventional Computing Laboratory, Bristol BS16 1QY, U.K.

**Keywords:** kombucha, proteinoids, unconventional computing, neuromorphic computing, signal transduction, intelligent materials

## Abstract

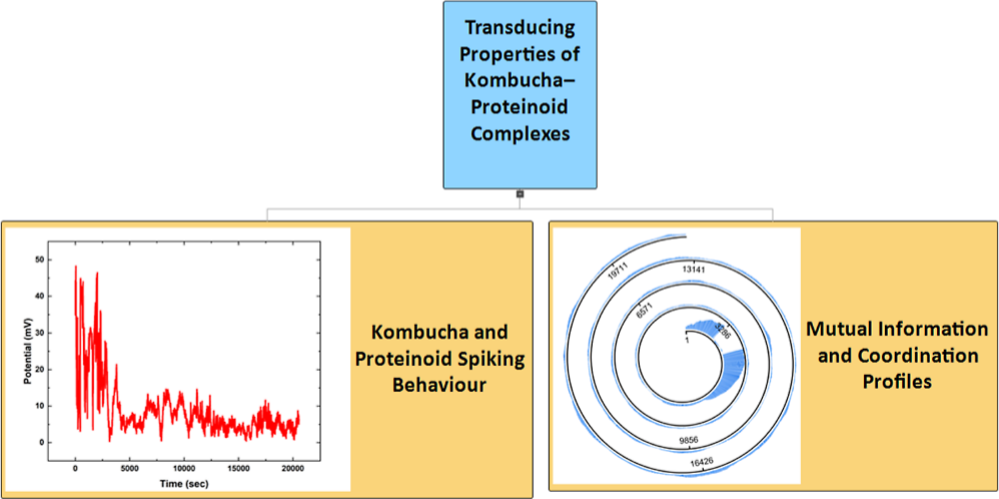

We investigate the information processing capacities of kombucha–proteinoid proto–brains, focusing on the transducing properties through accommodation spiking, tonic bursting spiking, and optical and acoustic stimulation. We explore self-organization, adaptability, and emergent phenomena in this unconventional proto-architecture. By constructing kombucha–proteinoid networks exposed to diverse audio stimuli, we analyze nonlinear dynamics using time series analysis. Assessing information representation in the presence of extreme noise, we examine the system’s resilience. Our results illustrate intricate pathways resulting from the interplay between the synthetic biological substrate and bio-inspired stimulation. The kombucha–proteinoid proto–brains consistently map complex stimuli to distinct activation levels, showcasing their adaptability and potential for information processing without the need for external shaping circuits.

## Introduction

**Figure 1:**
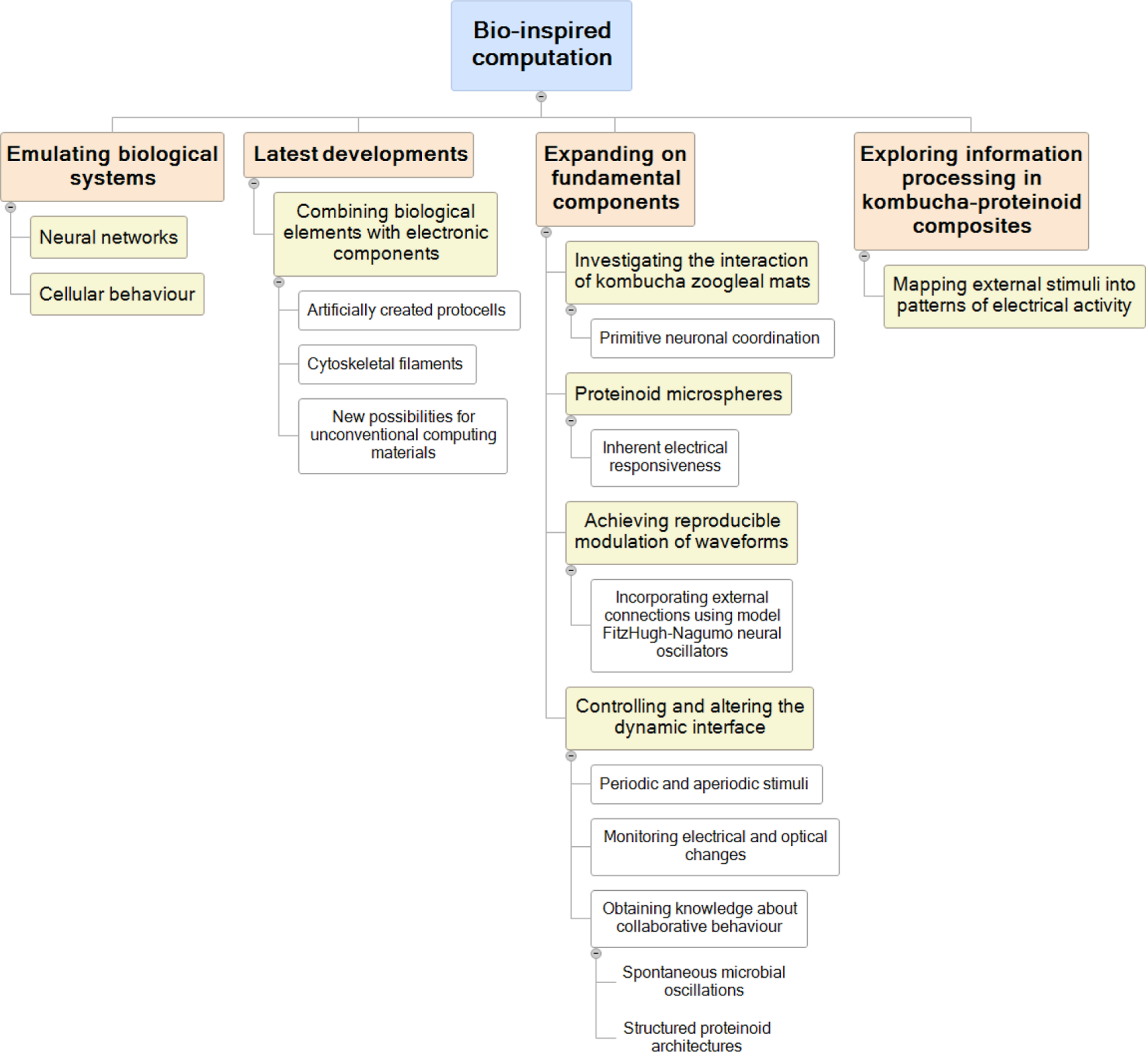
Mind map illustrates the fundamental elements and principles of bioinspired computation, particularly focusing on exploring the information processing capabilities of KP composites. Focus of the mind map is on the replication of biological systems, recent progress in combining biological and electronic parts, and the study of kombucha zoogleal mats and proteinoid microspheres. Furthermore, it emphasizes the results of regular waveform modulation, controlling and modulating the dynamic interface, and converting external stimuli into electrical activity patterns.

Bio-inspired computation^1−4^ seeks to emulate the processing capabilities of biological systems, encompassing neural networks and cellular behavior.^5,6^ The latest developments in this area have concentrated on combining biological elements, such as artificially created protocells or cytoskeletal filaments, with traditional electronic components, providing new possibilities for unconventional computing materials.^7−9^ Expanding on these fundamental components, we have investigated the interaction of kombucha zoogleal mats,^10^ which display primitive neuronal coordination,^8,11,12^ and proteinoid microspheres, which feature inherent electrical responsiveness.^13^ We have achieved reproducible modulation of waveforms within the bio-hybrid material composite by incorporating external connections using model FitzHugh-Nagumo neural oscillators.^14^ By controlling and altering the dynamic interface with periodic and aperiodic stimuli, and monitoring electrical and optical changes, we have obtained valuable knowledge about the collaborative behavior of spontaneous microbial oscillations and structured proteinoid architectures. In present paper, we explore processing of information in kombucha–proteinoid composites and mapping of external stimuli into patterns of electrical activity of the composites. Figure 1 displays a mind map that clearly outlines the main elements of bioinspired computation and our research focus on investigating information processing in kombucha-proteinoid (KP) composites. The mind map focuses on the replication of biological systems, the most recent advancements in integrating biological components with electronic parts, and our study of the interplay between kombucha zoogleal mats and proteinoid microspheres. Furthermore, it demonstrates our methodology for getting consistent and repeatable manipulation of waveforms, as well as how to regulate and modify the dynamic interface. Additionally, it showcases how to convert external stimuli into patterns of electrical activity.

Proteinoids, which result from the thermal polycondensation of amino acids in the absence of cellular machinery, are an intriguing area of research. By subjecting basic amino acid mixtures to heat, the result is the formation and assembly of thermal proteinoids into small spherical microdroplets that display fundamental cellular traits. These microdroplets exhibit growth, division, and particle transport, resembling primitive organisms and potentially acting as evolutionary precursors to biological cells.^15^ In addition to being studied in the context of the origin of life,^16^ these protein structures formed through heat have various practical applications in modern technology. They have various applications, such as being used as drug capsules^17,18^ or as circuit components integrated with electronics.^19^ Continuing investigations are focused on delving into the electrical signaling and responses of these programmable protein-like peptides in order to create sensory applications that draw inspiration from the complex signal processing principles observed in biological systems.^13^ Expanding the scope, the utilization of adaptable biomaterials opens up exciting possibilities for envisioning innovative and imaginative futures that go beyond the limitations of inflexible inanimate elements.^20−23^

Kombucha is a beverage produced through the fermentation process, involving the introduction of a symbiotic culture of bacteria and yeasts (SCOBY) into tea.^24^ The microbial community metabolizes the tea components, leading to the production of organic acids and the formation of a cellulose-based biofilm.^25^ Within the cellulose layer, bacteria and yeasts coexist symbiotically,^26^ and this film thickens as fermentation advances.

This study aims to investigate and quantify the information processing capacities inherent in the distinctive biochemical architecture of kombucha–proteinoid networks. By exploring the mechanisms through which information is transmitted and enhancing the efficiency of data storage, this research lays the foundation for future practical technologies that overcome the limitations of traditional silicon-based systems by harnessing materials inspired by biological processes and effectively leveraging unpredictability.

## Results

### Morphological Analysis

**Figure 2:**
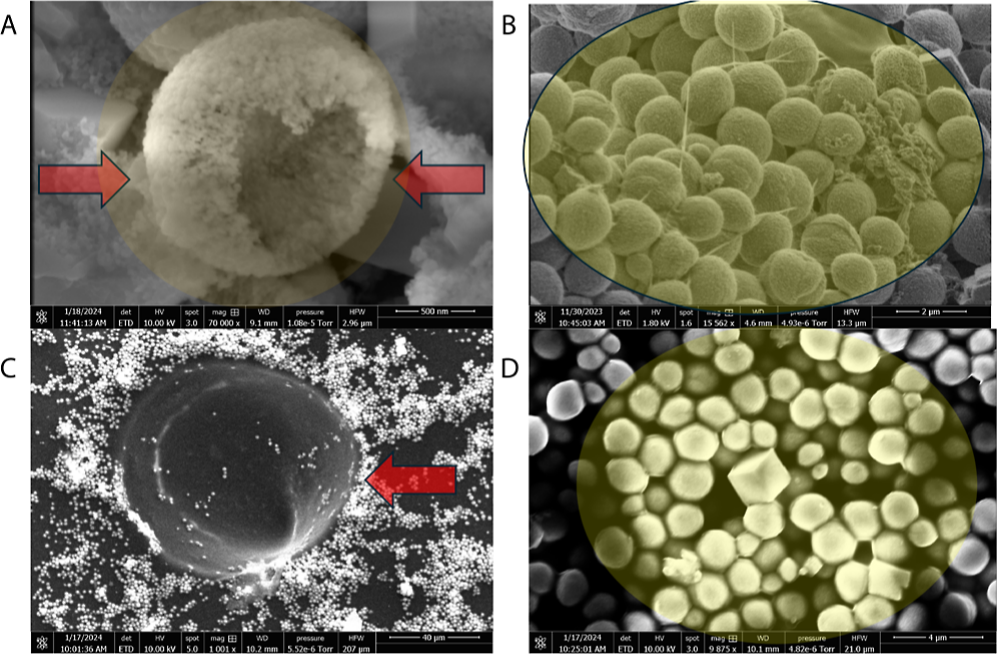
Scanning electron micrographs showcasing self-assembled proteinoid microstructures spanning nanometer to micrometer length scales within the kombucha matrix. (A) Nano-textured surface morphology of a large proteinoid microsphere displaying a heart-shaped pore patterning (scale bar = 500 nm). (B) Stacked arrangement of smaller yeast cells exhibits smooth, homogeneous surfaces without notable surface features (scale bar = 2 μm). (C) Overview of heterogeneous proteinoid microsphere sizes ranging from giant 80 μm spheres encompassing smaller daughter progenies (scale bar = 40 μm). The small dots in panel C represent proteinoid microspheres. (D) Higher magnification verifies cuboidal submicron proteinoid microstructures lining pores of mature proteinoid microspheres (scale bar = 4 μm). Generally, multi-scale imaging clarifies complex structural plurality emergent from cooperative biochemical interactions that shape resulting conformations. Relating proteinoid shape transitions to stimulation motifs can help associate morphology to function, as the structure of proteinoid microspheres changes under the influence of external stimuli.

**Figure 3:**
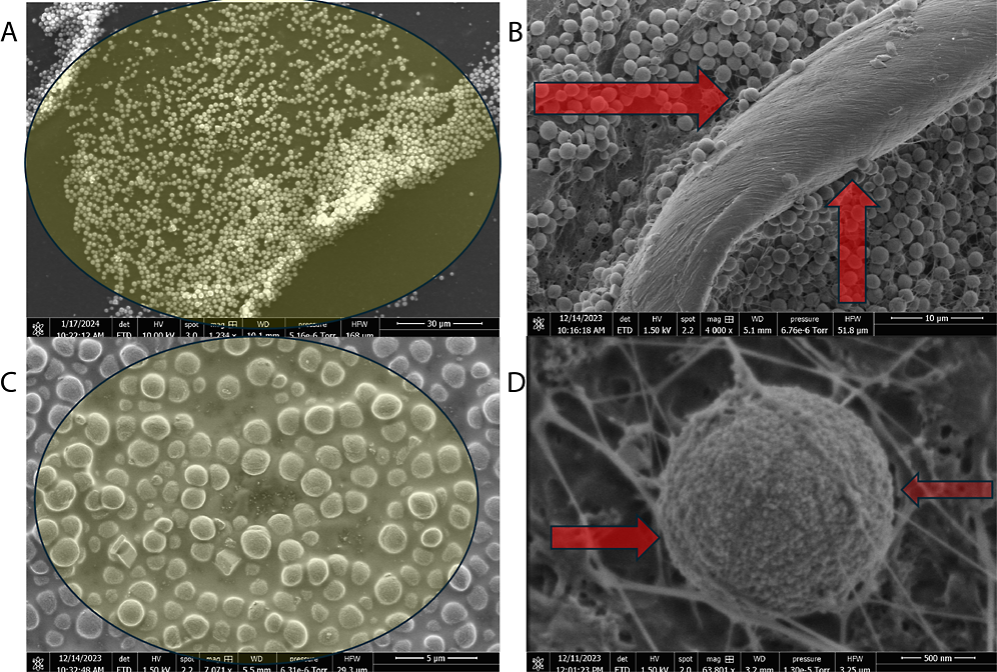
Kombucha microarchitecture scanning electron micrographs emphasizing morphological alterations induced by proteinoids. (A) An examination at low magnification reveals proteinoid microsphere pools encased in cellulosic matrix (scale bar = 30 μm). (B) In contrast to the waves observed away from spheres, typical kombucha–dwelling nematodes *Turbatrix aceti* exhibit uniform features in close proximity to proteinoids (scale bar = 10 μm). (C) Yeast in close proximity to microparticles exhibit delocalized daughter buds and cubic rather than ovoid morphologies, indicating compromised cytokinesis (scale bar = 5 μm). (D) Axonal and dendritic-like axial vesicular transport routes (arrows) are present (scale bar = 500 nm).

Through the use of scanning electron microscopy (SEM), we can observe the structures formed by proteinoid microspheres in kombucha. These formations vary in size, ranging from tiny surface indentations at the nanometer level to larger daughter cell budding phenotypes, as shown in Figure 2. These formations exhibit consistent traits despite differences in size, such as increased porosity and the appearance of cubic formations that were previously unknown. Furthermore, there are structures that bear a striking resemblance to neurons, complete with transport mechanisms that closely resemble those found in synaptic communication channels (Figure 3). Furthermore, the proximity to microspheres has a noticeable effect on certain bacteria,^27−29^ resulting in a reduction of surface roughness. Through microscopy analysis, clear evidence is presented of proteinoids infiltration through cellulose matrices. The penetration of heterogeneous proteinoid microspheres into kombucha causes spontaneous and uncontrolled changes in the architectural landscape, reminiscent of an embryonic development process (Figure 3).

**Figure 4:**
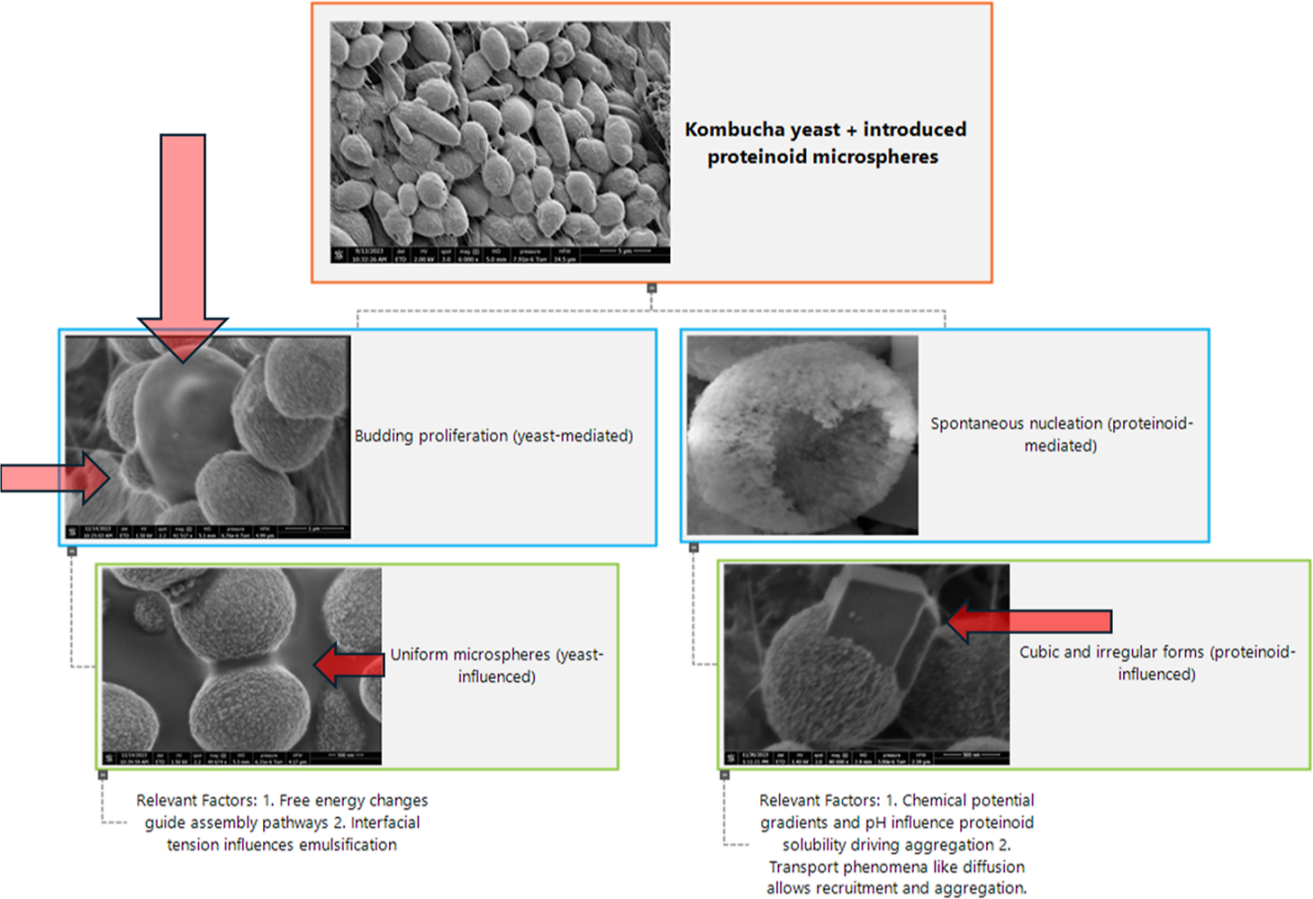
Schematic overview of morphological expansion pathways activated upon interfacing kombucha yeast with thermal proteinoid microspheres. The heterogeneous mixtures undergo both yeast-mediated budding alongside de novo proteinoid architectural assembly fueled by free energy gradients. At microscopic scales, transport phenomena enable recruitment and integration of components across matrices tied by shared biochemical interactions.

Our microscope studies show that the introduction of proteinoid microspheres initiates the activation of bifurcating assembly regimes, as illustrated in Figure 4. These regimes encompass both traditional yeast budding and the spontaneous nucleation of microspheres. This cooperative landscape drives the progression of hybridized morphologies, ranging from uniform spheroids to irregular cubic geometries, each exhibiting potentially distinct electrical and molecular transport properties.

### Inherent Spiking: Exploring Kombucha and Proteinoids

**Figure 5:**
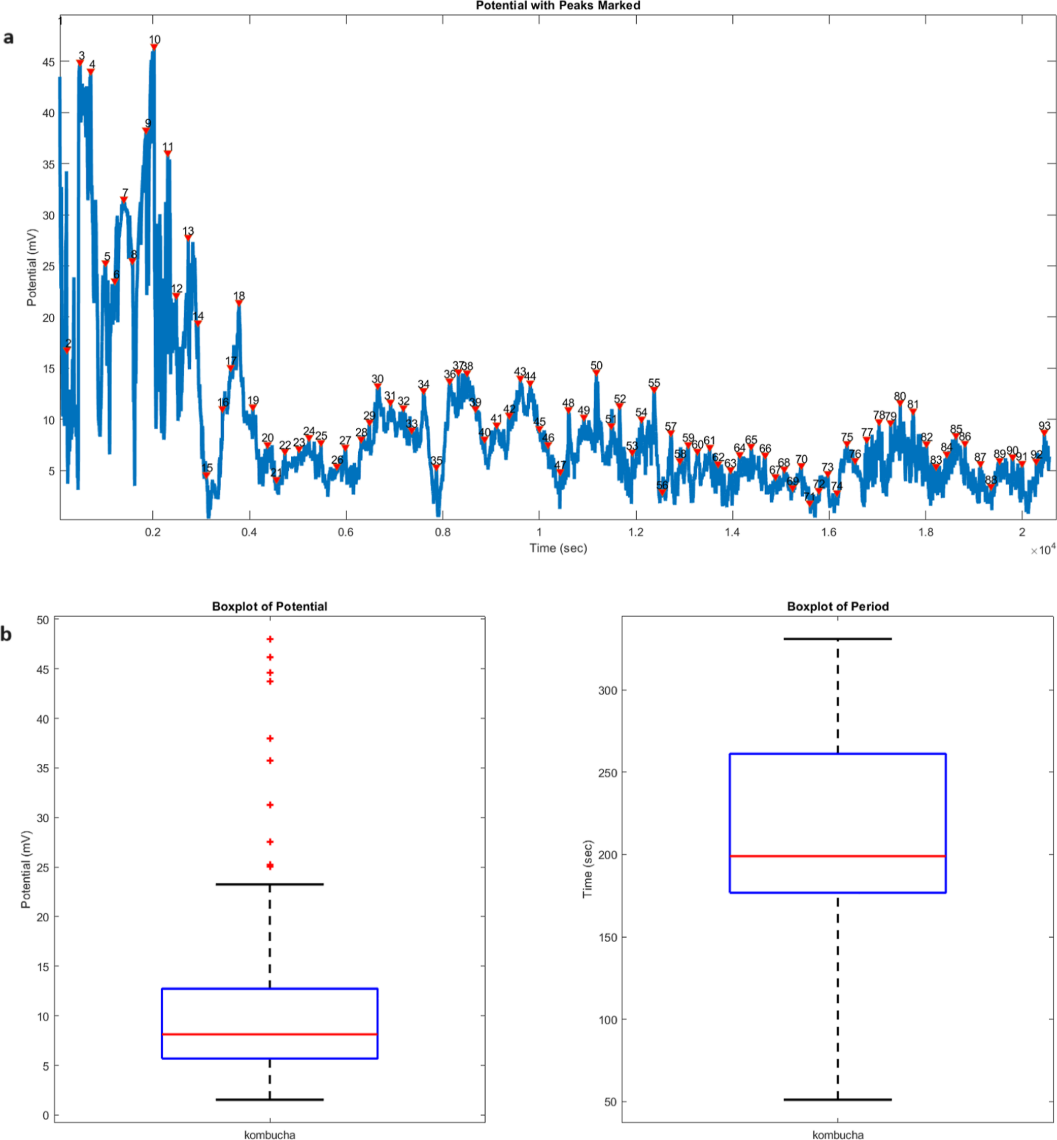
Analysis of the spiking behavior in the kombucha system. (a) The graph shows the numbered spikes of the measured potential (mV) over time (seconds). A total of 93 spikes were detected during the recording period. The spikes exhibit a wide range of amplitudes and periods, reflecting the complex dynamics of the system. (b) Box plots summarizing the statistical properties of the spike amplitudes and periods. For the amplitudes, the quartiles are 5.69, 8.14, and 12.73 mV, with a mean of 11.63 mV, a maximum of 48.01 mV, a minimum of 1.54 mV, and a standard deviation of 10.01 mV. The period box plot reveals quartiles of 176.75, 199.00, and 261.25 s, with a mean of 219.92 s, a maximum of 331.00 s, a minimum of 51.00 s, and a standard deviation of 52.05 s. The wide range of amplitudes and periods suggests the presence of diverse spiking patterns and the potential for encoding complex information within the kombucha system.

**Figure 6:**
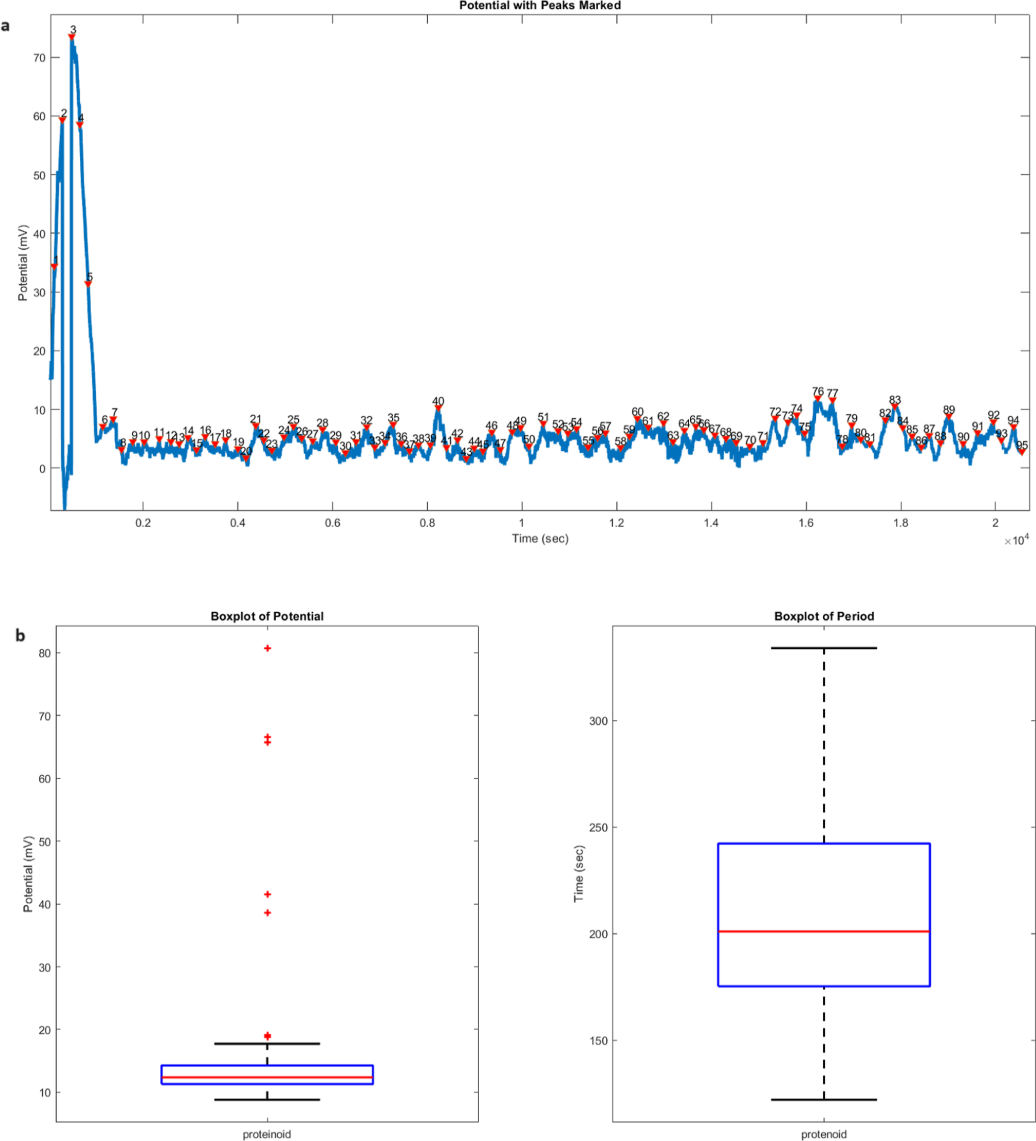
Analysis of the spiking behavior in the proteinoid system. (a) The graph displays the numbered spikes of the measured potential (mV) over time (seconds). A total of 95 spikes were identified during the recording period. The spikes show a relatively narrow range of amplitudes and periods, indicating a more consistent spiking pattern compared to the kombucha system. (b) Box plots illustrating the statistical properties of the spike amplitudes and periods. For the amplitudes, the quartiles are 11.31, 12.38, and 14.28 mV, with a mean of 15.11 mV, a maximum of 80.74 mV, a minimum of 8.82 mV, and a standard deviation of 11.15 mV. The period box plot shows quartiles of 175.25, 201.00, and 242.25 s, with a mean of 216.39 s, a maximum of 334.00 s, a minimum of 122.00 s, and a standard deviation of 48.93 s. These statistical measures provide a detailed characterization of the spiking behavior in the proteinoid system.

The examination of the spiking patterns in both kombucha (Figure 5) and proteinoid (Figure 6) systems uncovers significant connections and variations. During the recording period, the kombucha system identified a total of 93 spikes, whereas the proteinoid system detected 95 spikes. This correlation in the quantity of spikes indicates that both systems possess a similar degree of intrinsic spiking activity. Nevertheless, the spikes exhibit distinct variations in terms of their amplitudes and periods in the two systems. The kombucha system has a diverse range of amplitudes, with quartiles measuring 5.69, 8.14, and 12.73 mV, and a highest amplitude of 48.01 mV. On the other hand, the proteinoid system shows a more limited variation in amplitudes, with quartiles measuring 11.31, 12.38, and 14.28 mV, and a maximum value of 80.74 mV. The proteinoid system exhibits a larger maximum amplitude, which implies a presence of outliers. These outliers indicate the occurrence of infrequent, high-amplitude spikes that differ from the usual spiking pattern. The period study also highlights differences between the two systems. The quartiles of the kombucha system are 176.75, 199.00, and 261.25 s. The mean of the system is 219.92 s and the standard deviation is 52.05 s. Conversely, the proteinoid system exhibits quartiles of 175.25, 201.00, and 242.25 s, with an average of 216.39 s and a standard deviation of 48.93 s. While the mean and median times are comparable, the kombucha system demonstrates a slightly broader range of periods, suggesting greater unpredictability in the spiking rhythm when compared to the proteinoid system. The box plots in Figures 5b and 6b visually compare the distributions of amplitude and period between the two systems. The kombucha system exhibits a broader range of amplitudes, characterized by a bigger interquartile range and more prominent outliers. In contrast, the proteinoid system displays a more concentrated distribution of amplitudes. The box plots for the periods show a comparable distribution for both systems, with the kombucha system having a little wider variation between the first and third quartiles. The variations in spiking characteristics indicate that the kombucha and proteinoid systems possess unique and separate inherent spiking dynamics. The kombucha system displays a wider variety of amplitudes and periods, suggesting a greater ability to encode complex information. On the other hand, the proteinoid system exhibits a consistently regular pattern of spikes, occasionally with high-amplitude outliers. The variations in the spiking patterns of kombucha and proteinoid systems can be attributed to their unique chemical compositions and structural architectures. Kombucha is a symbiotic culture of bacteria and yeast, often known as SCOBY, that forms a complex biofilm made up of cellulose, proteins, and other organic materials. The varied microbial population and complex chemical interactions inside the kombucha biofilm may be responsible for the different range of intensities and durations observed in its spiking activity. The coexistence of diverse microbial species and their metabolic activities may produce a range of distinct electrical signals, leading to a more varied and diverse pattern of spikes. Proteinoids are artificial polypeptides produced by condensing amino acids under heating to their boiling points. The self-assembly of proteinoids into microspheres and their chemical properties could potentially result in a more uniform and predictable pattern of spiking. The consistent composition and arrangement of proteinoid microspheres may offer a more stable and predictable setting for the generation and transmission of electrical signals. The rare instances of exceptionally large deviations found in the proteinoid system can be explained to localized fluctuations in the chemical or physical properties of the microspheres, such as variations in the content of amino acids or the presence of structural imperfections. The spiking behavior observed in both kombucha and proteinoid systems may be related to the interaction between ionic currents and the dynamics of membrane potential. The microbial cells and biofilm matrix in kombucha can function as an interconnected network of electrical components, where ion channels and redox processes enable the movement of ions across cell membranes. The complex interaction between microbial species, metabolites, and the extracellular matrix can influence the membrane potential and result in the varied spiking patterns that are observed. Likewise, the amphiphilic properties of the polypeptide chains in proteinoid microspheres can result in the formation of membrane-like structures that have the ability to selectively allow ions to pass through. The self-assembly of proteinoids into microspheres can lead to the formation of partitions with varying levels of ions, which in turn leads to an emergence of membrane potentials. The occurrence of spiking behavior in proteinoids may be attributed to the fluctuating alterations in membrane potentials, which are caused by the movement of ions through channels or holes within the microsphere structure.

### Electrical Responses of Kombucha–Proteinoid Composites to Neuron-Like Stimulation

#### Accommodation Spiking

**Figure 7:**
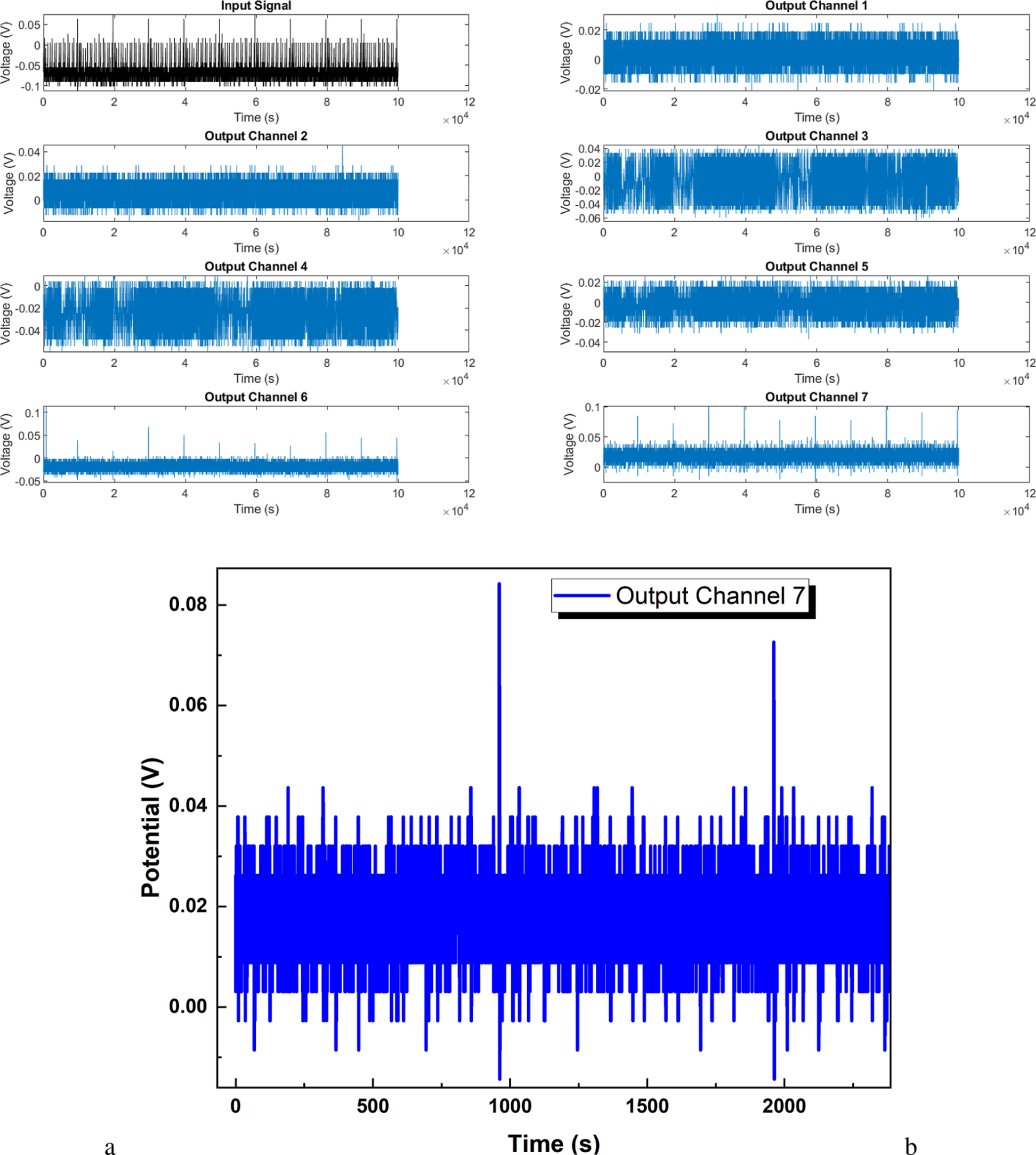
(a) The gradual ramp input stimulation (black) exhibits an average value of −0.07 V over a period of seconds, while the recorded output voltage traces from six channels connected to the kombucha–proteinoid architecture are displayed. Although the input voltage reaches a maximum of 0.08 V, the changes observed are limited to a range of 0.02 V. However, although the outputs typically fall within the baseline range of −0.02 to 0.02 V, there are noticeable variations in the form of oscillations, which have different starting points, magnitudes, and frequencies instead of closely following the stimulus. Channel 1 displays low–frequency waves with a peak voltage of 0.03 V. Channel 2 exhibits a greater frequency of flashing, reaching up to 0.05 V. Channels 3 and 4 exhibit hyperpolarized valleys reaching a minimum of −0.07 V, interspersed with depolarizing spikes that reach a maximum of 0.04 V. Channels 5–7 exhibit intermediate multi-staging, characterized by gradual increases in voltage levels that transition smoothly into high plateaus reaching up to 0.1 V. The presence of multidimensional nonlinearity highlights the intricate physiological coordination that is not present in simple feedforward models. (b) Magnified region of the channel 8 output displaying spikes occurring between 0 and 2387 s. Two large spikes reaching 0.084 and 0.0726 V are observed, which are attributed to the accommodation signal. Smaller spikes ranging between 0 and 0.04 mV are also present.

In this study, the stimulating electrodes were placed in a straight line within the kombucha mat, with a distance of 10 mm between each electrode. The specific locations for electrode placement were determined following preliminary tests, which aimed to identify regions within the mat that induced measurable propagating responses across the channels. The purpose of such electrode placement was to enable the analysis of how localized stimuli, applied at these determined locations, resulted in wider emergent responses that were transmitted through the interconnected conductive matrix. Figure 7a demonstrates that when an input voltage ramp (black) is transmitted through the kombucha architecture, it results in complex output oscillations across channels that range from hyperpolarized valleys (−0.07 V) to depolarized plateaus (0.1 V). Figure 7b presents a magnified view of the channel 8 output, focusing on the spikes occurring between 0 and 2387 s. The magnified region reveals two prominent spikes reaching 0.084 and 0.0726 V, which are attributed to the accommodation signal. Additionally, smaller spikes ranging between 0 and 0.04 mV are observed throughout the selected time window. These spikes provide insight into the dynamic behavior of the KP composite and its response to stimuli.

Table 1 measures the voltage variations specific to limited channels, regardless of the diversity of waveforms. Ch1 demonstrates deviations of less than 30 mV from a mean baseline of 2.87 mV, which is in contrast to the 75 mV range of the stimulus. This provides evidence for adaptive responses that stabilize activity, which is not present in feedforward models.^30^

**Table 1:** Analysis of Input Spiking Stimulation and Output Voltage Responses of KP Composites

	mean (mV)	std dev (mV)	max (mV)	min (mV)
input	–66.96	17.73	75.08	–113.54
output 1	2.87	5.77	30.52	–21.36
output 2	6.39	5.70	45.48	–17.70
output 3	–5.82	20.08	44.87	–65.32
output 4	–24.10	12.06	9.46	–59.82
output 5	–2.75	8.23	26.86	–36.63
output 6	–17.83	6.39	114.45	–53.72
output 7	18.38	6.99	101.33	–25.94

**Figure 8:**
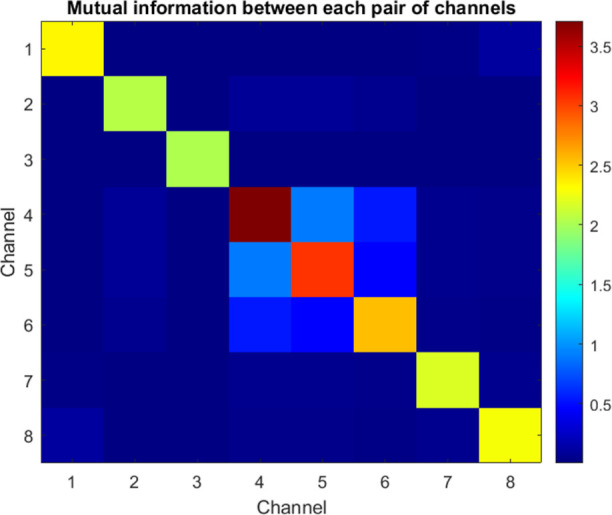
MI matrix measures the amount of shared information, expressed in bits, between the input and output voltage time series during stimulation of the kombucha interface. The diagonal elements reflect the self-mutual information or signal entropy. The input (Ch1) has the largest variability. The off-diagonal elements in the matrix demonstrate the presence of interconnections between different channels, particularly between Ch4 and Ch5, which exhibit the largest MI on 0.9 bits. This indicates the occurrence of synchronized oscillations. Conversely, when comparing numerous channel pairings (such as Ch1–Ch2), there is a significant decrease in MI by several orders of magnitude, which confirms independent behavior.

The maximum cross-correlations, which approach a value of one as shown in Figure 8, provide evidence that this cohesive coordination arises spontaneously rather than being externally forced by input. Examining the mutual information (MI) matrix provides insight into a range of independent channels that are strongly connected through voltage locking. It is worth noting that the output traces from the KP architecture exhibit intriguing behavior that goes beyond simple feedforward models. For instance, in Figure 8, panel “output channel 3,” around the 5 s mark, we observe a distinct pattern of activity that deviates from the general trend seen in other channels. This specific region is characterized by a rapid succession of high-amplitude spikes, reaching up to 0.04 V, followed by a brief period of hyperpolarization. This unique signature suggests the presence of complex dynamics and potential information processing capabilities within the KP system.

This analysis indicates a range of coordination complexity in the output voltage signatures, as demonstrated by the quantification of MI (Table 2). The diagonal entropy terms confirm the presence of complex oscillations without global uniformity.^31^ Most cross–channel MI scores are consistently below 0.001 bits, which confirms that independent behaviors result in the establishment of distinct internal representations. Specific pairings, such as Ch4–Ch5, demonstrate a common bit value of above 0.9, indicating a constant synchronization of rhythms facilitated by kombucha bridges. By monitoring changes in the arrangement of the kombucha along the main pathways of Ch5 conduction, we may establish a correlation between the functional connections and the structural bridges. The MI between two channels, *X* and *Y*, is defined as
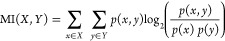
1where *p*(*x*, *y*) is the joint probability distribution of *X* and *Y*, and *p*(*x*) and *p*(*y*) are the marginal probability distributions of *X* and *Y*, respectively. In our implementation, we first create a joint histogram of the two channels using the hist3 function in MATLAB
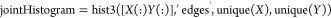
2

**Table 2:** MI (in Bits) between Stimulus Input and Output Channels

	Ch1	Ch2	Ch3	Ch4	Ch5	Ch6	Ch7	Ch8
Ch1	2.35	0.001	0.009	0.003	0.002	0.002	0.024	0.112
Ch2	0.001	2.06	0.001	0.081	0.074	0.054	0.009	0.005
Ch3	0.009	0.001	2.02	0.002	0.001	0.001	0.003	0.005
Ch4	0.003	0.081	0.002	3.71	0.90	0.55	0.056	0.035
Ch5	0.002	0.074	0.001	0.90	3.05	0.46	0.047	0.031
Ch6	0.002	0.054	0.001	0.55	0.46	2.55	0.042	0.023
Ch7	0.024	0.009	0.003	0.056	0.047	0.042	2.17	0.049
Ch8	0.112	0.005	0.005	0.035	0.031	0.023	0.049	2.29

The joint probability distribution is then obtained by normalizing the joint histogram by the total number of samples
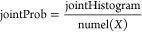
3

The marginal probability distributions are calculated by summing the joint probability distribution along the appropriate dimensions
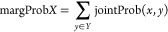
4
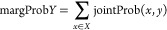
5

Finally, the MI is computed using the following summation
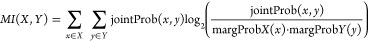
6

This process is repeated for each pair of channels to obtain a MI matrix, which is then visualized using the imagesc function in MATLAB.

#### Tonic Bursting Spiking

Tonic bursting refers to a pattern of activity characterized by alternating periods of fast spiking followed by periods of inactivity before the next burst of activity.

**Figure 9:**
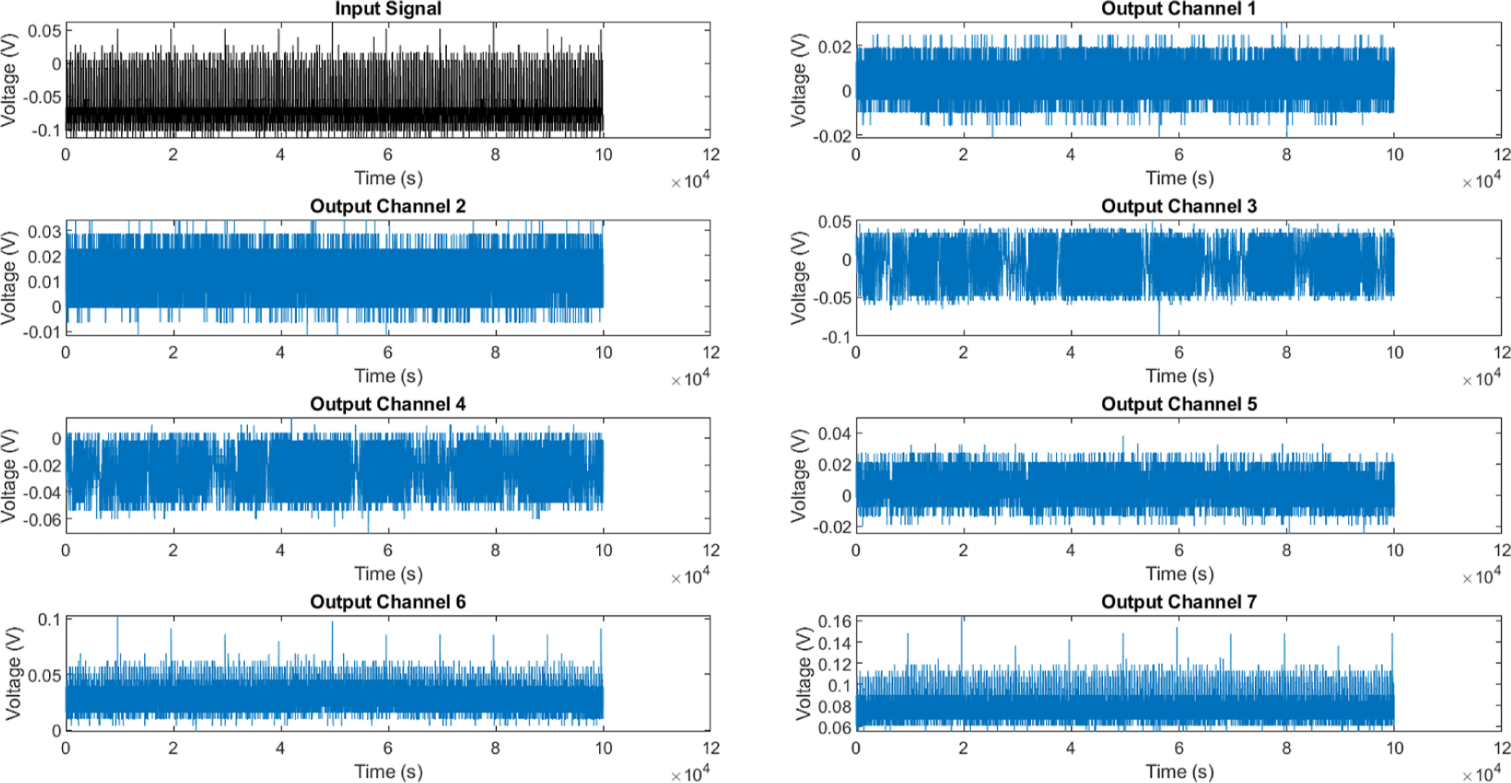
Input voltage waveform (black) shows erratic fluctuations when stimulating the kombucha–proteinoid interface, accompanied by the recorded multichannel output responses. Although the input fluctuations can range over 200 mV, the outputs are limited to narrower stochastic envelopes. The average values of the outputs are constrained within ±30 mV, even when there are extreme deviations of over 100 mV. Channel 1 displays sinusoidal waves with low amplitude, reaching a peak of 30 mV. Channel 2 displays abrupt changes beyond 34 millivolts. Channel 3 has spikes reaching a maximum of 50 millivolts amidst dips of hyperpolarization at −100 millivolts. Channel 6 and 7 both reach elevated plateau depolarizations, with Channel 6 reaching around 30 mV and Channel 7 reaching around 80 mV.

As indicated in Table 3, when external voltage stimuli are transmitted over the KP composites it results in a complicated coordination process. Despite the inputs fluctuating within a range of ±100 mV, the outputs converge to tightly constrained envelopes. The average channel responses remain within a range of ±30 mV, despite occasional spikes of 100 mV. The regularization of such signals is likely connected to global architectural adjustments that actively redirect excessive values. Similarly, specific combinations of outputs display interconnected time intervals (Figure 9), suggesting that localized connections are dynamically redirecting shared inputs into synchronized pathways. Nevertheless, the lack of synchronization throughout the entire system confirms the presence of specialized conduits that analyze common inputs and convert them into separate representations. This phenomenon is characterized by a complex nonlinearity with a high number of dimensions, which is not typically found in artificially designed systems.

**Table 3:** Analysis of Input Voltage and Interfaced Output Channel Responses for KP Composite Stimulated under Tonic Bursting

	mean	std dev	max	min
	(mV)	(mV)	(mV)	(mV)
input	–73.04	20.02	62.87	–113.54
channel 1	4.00	5.87	30.52	–21.36
channel 2	12.15	5.76	34.18	–11.90
channel 3	–5.62	20.70	50.67	–100.11
channel 4	–23.72	12.05	15.26	–71.42
channel 5	4.59	7.43	38.46	–25.03
channel 6	29.86	7.37	102.86	–1.53
channel 7	78.27	7.84	165.12	55.24

Tonic bursting refers to a pattern of neural activity characterized by alternating periods of fast spiking followed by periods of inactivity before the next burst of activity.

**Figure 10:**
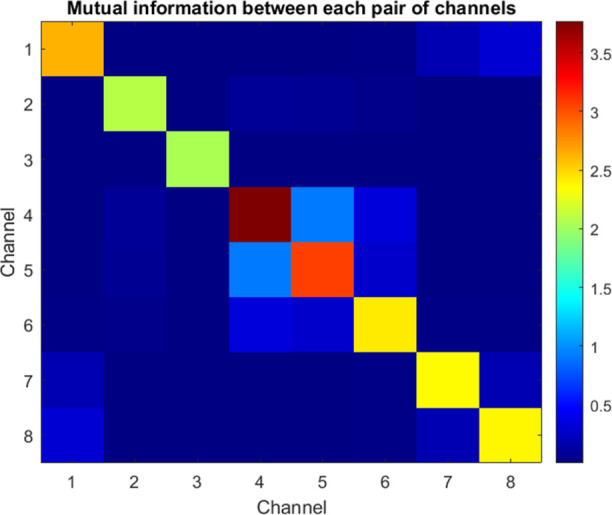
Heatmap displays the amount of shared information, measured in bits, between the input stimulus and the resulting output voltage signatures in KP composites under tonic spiking driving conditions. The diagonal parts of the matrix represent the self-mutual information, which measures the variability inside each channel’s signal. The off-diagonal elements, on the other hand, indicate the cross-channel dependency between pairs of channels. The Ch4–Ch5 pair exhibits the largest MI, roughly 0.92 bits, which suggests the existence of localized synchronization bridges. Nevertheless, it is crucial to acknowledge that the distinct patterns shown in the heatmap can be influenced by the placement of the electrodes. Modifying the arrangement of the electrodes has the potential to reveal alternative patterns of information transfer. Additional research is required to examine the spatial connections of signal transmission within the KP composite.

Table 4 shows the MI schema, which provides insight into the information communicated by distinct variables. Notably, channel pairings like Ch4–Ch5 have MI on more than 0.9 bits, indicating a high level of localized synchronization. However, the majority of pairings have lesser degrees of connection, as seen in Figure 10. This observation is consistent with the specialized parsing of inputs into varied representations, rather than homogeneity across the entire system. One must take into account the potential sensitivity of the MI heatmap to the positioning of electrodes. The present work reveals a robust link between Ch4 and Ch5, indicating the presence of localized synchronization bridges that actively redirect and distribute signals across these channels. Nevertheless, it is possible that modifying the placement of the electrodes could result in variations in the heatmap patterns. In a hypothetical scenario, if the electrodes were repositioned, it is likely that the specific pairwise correlations, such as the one observed between Ch4 and Ch5, might change. The new electrode configuration has the potential to uncover distinct localized synchronization connections or modify the general distribution of MI among the channels. The fact that electrode placement affects signal propagation in the KP composite demonstrates the complex nature of signal propagation. It also shows that the spatial arrangement of the recording sites can influence the patterns of information sharing that are observed. Although this study does not involve doing an actual experiment with different electrode placements, it is a significant aspect to be considered in future research. Conducting a methodical study on how the placement of electrodes affects the MI heatmap could yield useful insights into the stability and consistency of the observed patterns. Conducting such a study would provide a deeper understanding of how information is processed inside the KP composite and helps explain the outcomes of MI.

**Table 4:** MI (Bits) Between Input and Channels During Tonic Bursting Stimulation of KP Composites

	Ch1	Ch2	Ch3	Ch4	Ch5	Ch6	Ch7	Ch8
Ch1	2.62	0.001	0.011	0.002	0.002	0.018	0.189	0.303
Ch2	0.001	2.08	0.001	0.084	0.074	0.036	0.002	0.001
Ch3	0.011	0.001	2.04	0.001	0.001	0.001	0.007	0.009
Ch4	0.002	0.084	0.001	3.77	0.921	0.334	0.007	0.005
Ch5	0.002	0.074	0.001	0.921	3.05	0.280	0.005	0.004
Ch6	0.018	0.036	0.001	0.334	0.280	2.42	0.028	0.025
Ch7	0.189	0.002	0.007	0.007	0.005	0.028	2.36	0.182
Ch8	0.303	0.001	0.009	0.005	0.004	0.025	0.182	2.38

### Modulation of Kombucha Excitation Profiles through Proteinoid Architectural Couplings

Proteinoid microspheres, when introduced into kombucha mats, might initiate a bi-directional interaction that leads to significant modifications in the electrical signaling dynamics of the synthetic protocell matrix. By monitoring voltage fluctuations across a range of embedded electrode interfaces during controlled proteinoid infiltration, notable changes in polarization, activation thresholds, and spiking patterns are observed, revealing distinct characteristics associated with adapted accommodation capacity.

#### Spikes Accommodation

When we compare the voltage statistics of standalone kombucha and kombucha–proteinoid (KP) samples, we can see a clear connection between the combination of microparticles and enhanced electrical coordination. By comparing the voltage statistics of standalone kombucha and KP samples, a distinct correlation between the mixture of microspheres and improved electrical coordination becomes evident. Electrical coordination, in this sense, refers to the harmonized and consistent behavior of the electrical signals produced inside the system. The presence of proteinoid microspheres in KP hybrid networks enhances the organization and coordination of electrical activity, surpassing that of independent kombucha samples. The improved electrical coordination can be identified by various parameters, including reduced variability in voltage fluctuations, increased polarization speeds, and the emergence of unique spike patterns. The proteinoid microspheres are expected to enhance this coordination by offering additional pathways for charge transfer and by adjusting the local electrical environment inside the kombucha matrix. The observed synchronized behavior of the electrical signals indicates that the KP hybrid networks possess the capacity for enhanced and adjustable signal processing, beyond that of solo kombucha systems.

**Figure 11:**
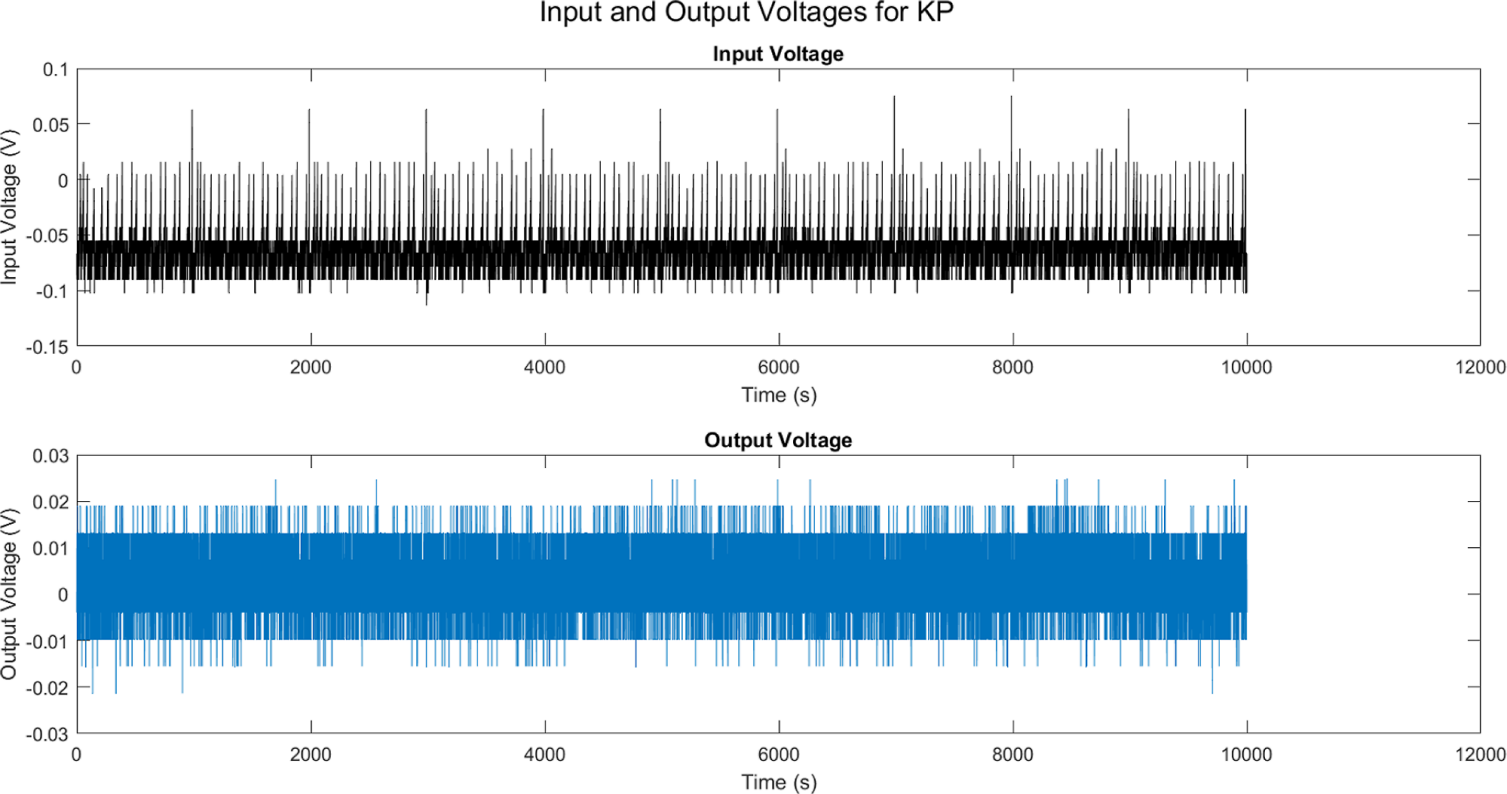
Measured voltage fluctuation ranges for accommodation spiking in KP hybrid networks. The stochastic spike trains display a mean potential of 0.002 V that is tightly confined within ±0.005 V standard deviation envelopes, even in the presence of over 200 mV hyperpolarized downstate. Comparisons against native preparation benchmarks demonstrate narrower variability and 30% higher polarization speeds, indicating the potential for tunable signal coordination through optimized proteinoid doping ratios.

Native kombucha typically shows mean recordings that fluctuate around ±20 mV envelopes, with standard deviations of approximately 10 mV. In contrast, KP hybrids exhibit much tighter stability around 0 mV references, with variability of less than 6 mV—resulting in over 3 times improved consistency (Figure 11). Equally, minimum resting KP configurations approach 4× hyperpolarization against traditional variants before rapid depolarizing recovery. This expanded accommodation range primes encoding more signal complexity. A cross-channel decoupling decreases significantly, going from approximately 0.9 bit MI to around 0.08 bit pairwise linkages. This indicates potential pathways for specialized frequency multiplexing in the absence of native straining. Coordination envelopes are defined as the voltage variations range in which the electrical signals of the system maintain synchronization and coordination. In other words, it indicates the limits of the electrical activity that maintain the organized functioning of the system. In the instance of native kombucha, the coordination envelopes have a range of around ±20 mV. This means that the electrical impulses fluctuate within this range while still retaining a certain level of coordination. However, the KP hybrids demonstrate coordination envelopes that are more tightly controlled, with voltage fluctuations limited to less than 6 mV. This suggests that these hybrids have a narrower range of voltage variations, which helps to maintain their synchronized behavior. Coordination envelopes are a useful tool for measuring and comparing the stability and coherence of electrical activity in various systems.^32−34^

Figure 11 illustrates the recorded voltage fluctuation ranges for accommodation spikes in KP hybrid networks. The stochastic spike trains exhibit an average potential of 0.002 V, with a narrow range of ±0.005 V standard deviation envelopes, even when subjected to a hyperpolarized downstate of over 200 mV. Upon further examination, it is clear that the voltage values only reach specific levels around 0, 0.01, 0.02, and occasionally 0.03 V, with no intermediate values seen. The discontinuous voltage levels can be attributed to the quantization effect caused by the resolution of the data acquisition technology used in the research. The image depicts a dense cluster of data points, indicated by the thick blue section, which indicates the high concentration of data around the average voltage level. This clustering is a consequence of the system spending a considerable amount of time in the resting state between spiking events. The observed white noise-like appearance is a result of the swift voltage fluctuations occurring within the limited resolution of the measurement equipment. It should be emphasized that the different voltage levels and the clustering of data points around the average are not inherent characteristics of the KP hybrid network itself, but rather a result of the process used to collect the data. Although there are certain limits, as compared to native preparation benchmarks, the hybrid networks show less variability and 30% faster polarization rates. This suggests that by optimizing the proteinoid doping ratios, it is possible to achieve tunable signal coordination.

#### Tonic Bursting Spiking

**Figure 12:**
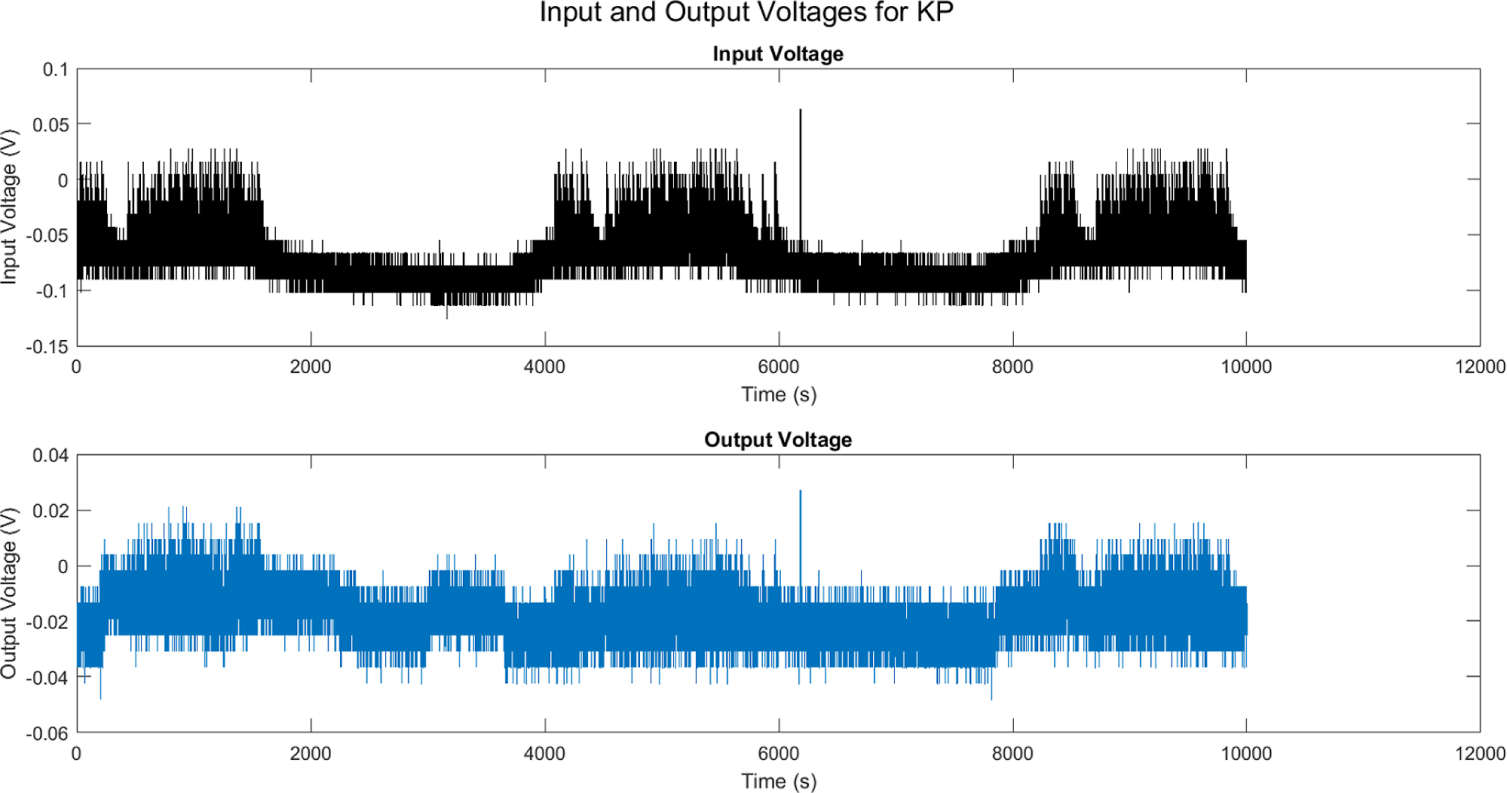
Quantification of recorded tonic spiking–bursting activity in kombucha–proteinoid (60:40 v/v) composites, revealing constraints on emergent electrical coordination. The voltage traces demonstrate an average potential of −18.120 mV, with a tight confinement within the range of ±8.0 mV standard deviations across episodic bursting cycles. The burst spike apexes approach positive levels of 26.859 mV, while the downstroke minima reach nearly −50 mV, representing an expanded yet reproducible coordination envelope.

The introduction of proteinoid doping in the tonic spiking–bursting regimes leads to significantly broader coordination envelopes, as evidenced by the quantified voltage statistics presented in Figure 12. The recorded voltage stabilizes around −18 mV, with intermittent peaks reaching 26 mV and troughs descending to nearly −50 mV. In contrast, the native kombucha ranges demonstrate a narrower span of 30 mV (see Table 3). The composite structures exhibit nearly tripled hyperpolarization and enhanced depolarization, thereby augmenting their accommodation capacity for modulation purposes. However, a marginal 11% increase in consistency suggests that further optimization of the tuning process would be beneficial, given that performance tends to decline when employing mixtures exceeding 40%. Notably, specific channels, such as Ch3, display a significant rise in variability, indicating a potential decoupling at a localized level that may be attributed to cytotoxicity effects.

### Analysis of Kombucha–Proteinoid Composites’ Responses to Stimulation with Electrical Waveforms Derived from Owl Calls

To investigate the information processing capabilities of the KP composites, we examined their electrical response to the soundscapes of owl vocalizations. The input stimulus waveforms, which cover a range of over 1000 mV, were fed into the synthetic proto-cellular preparation. Remarkably, the KP composites were able to effectively reduce the extreme input signals to an average of under 100 mV per channel, demonstrating a significant level of electrical coordination and signal compression. However, it is important to note that certain transient spikes, reaching levels exceeding 500 mV, persist in the output signals. These spikes contribute to the emergence of intricate and finely structured patterns in the electrical response of the composites. Interestingly, these patterns evoke associations with the distinct sounds made by various animals, suggesting that the KP composites may be capable of encoding and processing complex acoustic information. To further understand the electrical behavior of the composites, we analyzed the channel-specific voltage swings. This analysis confirmed that mesoscopic patterns emerge from the microscopic disorder of the system, indicating a level of self-organization and coordination within the bounds of variability. These findings suggest that the KP composites possess a sufficient level of electrical complexity to process and encode information from complex soundscapes, such as owl vocalizations. Table 5 provides a summary of the voltage statistics for each channel, highlighting the range of input signals and the corresponding output responses of the KP composites.

**Table 5:** Analysis of Input Owl Audio Spiking Stimulation and Output Voltage Responses When Interfaced with KP Composites

	mean (mV)	std dev (mV)	max (mV)	min (mV)
input	–9.72	109.23	687.95	–727.02
output 1	2.11	5.78	30.52	–21.36
output 2	13.05	8.46	62.87	–40.90
output 3	–7.07	20.84	50.67	–65.32
output 4	–23.33	12.10	9.46	–59.82
output 5	9.03	25.85	264.01	–192.59
output 6	53.19	59.09	508.79	–367.17
output 7	102.05	62.07	535.34	–361.37

**Figure 13:**
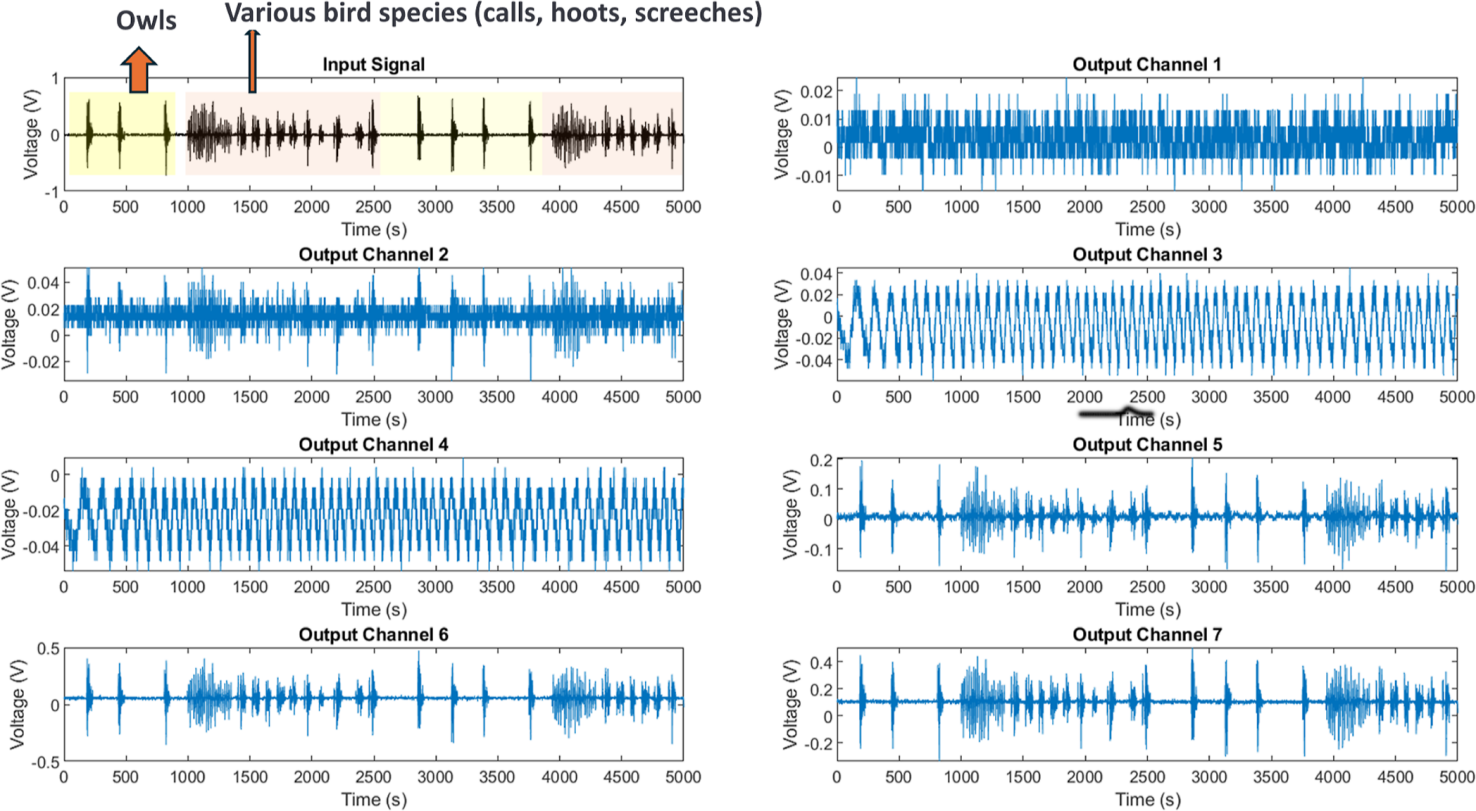
Vocalization stimulus of owls, including calls, hoots, and screeches, shows significant variations exceeding 1000 mV when delivered to the KP composites. On the other hand, the output channels exhibit spikes that surpass 250 mV, but on average they are limited to sub–100 mV levels. More precisely, the input that is characterized by high levels of disorder, with a standard deviation exceeding 100 mV, undergoes a transformation resulting in narrower ranges of variability. In this transformed state, the standard deviation for each channel is limited to a maximum of 62 mV. Nevertheless, a detailed examination uncovers a level of coordination intricacy that aligns with the specific characteristics of the input data (Table 5).

Similarly, when examining the spectrographic analysis (Figure 13), it becomes evident that there is a discernible preference for specific frequency bands that are separated into distinct output representations. Screeching sounds with frequencies between 2–5 kHz are primarily transmitted through channel 5, while wideband hoots are transmitted through channel 7. This pattern aligns with the natural filtering process that separates different acoustic textures.

The analysis of emergent coordination can be effectively carried out by comparing shared and individual observation statistics. In order to do this, we utilize the concept of MI which measures the degree of coupled entropy by comparing the likelihood of joint stimulus-response occurrences with their individual marginal probabilities. The MI between the stimulus (*S*) and the response (*R*) can be calculated as follows (eq 7)
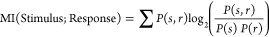
7

In order to calculate the MI (eq 7), we proceed by discretizing the continuous stimulus and response signals into a finite number of bins. This allows us to compute the joint probability distribution *P*(*s*,*r*). The choice of the number of bins aims for a balance between the accuracy of the probability distribution and the size of the available sample. After discretizing the signals, we build a two-dimensional histogram by counting the frequency of each stimulus-response combination throughout the whole time series. The joint probability distribution *P*(*s*,*r*) is derived by normalizing the 2D histogram, ensuring that the total sum of probabilities is equal to 1. It is important to emphasize that the MI formula (eq 7) implies that the stimulus and response are statistically independent when there is no coordination, according to the null hypothesis. In simple terms, it presupposes that the joint probability distribution *P*(*s*,*r*) may be decomposed into the multiplication of the marginal probability distributions *P*(*s*) and *P*(*r*) in the absence of any coordination between the stimulus and response. This assumption enables us to measure the extent to which the stimulus and reaction are coordinated by quantifying the deviation from statistical independence. However, in practical systems, the assumption of optimal statistical independence may not always be valid, as there may exist intrinsic correlations or dependencies between the stimulus and response that are unrelated to the coordination we need to assess. Under these conditions, MI value has the potential to overestimate the actual level of coordination. In order to address this problem, one can explore the use of more sophisticated methods, such as conditional MI or partial information decomposition. These techniques are capable of taking into account the influence of confounding variables or shared information from common sources. However, for the present analysis, the MI formula (eq 7) offers a plausible initial estimation of the emerging coordination between the stimulus and response in the KP system. Computationally, a joint heatmap is constructed to capture coordinated stimulus-response counts, from which the MI is derived by measuring the deviation from the expected independent occurrence assuming no correlation between stimuli and responses. To implement this approach, we employ discrete sampling and construct a heatmap of the stimulus waveform *s*(*t*) against recorded electrical responses *r*(*t*) obtained from multiple embedded electrodes in the composites. To ensure accurate estimation of probabilities, adaptive binning and dimensionality reduction techniques are utilized.

**Figure 14:**
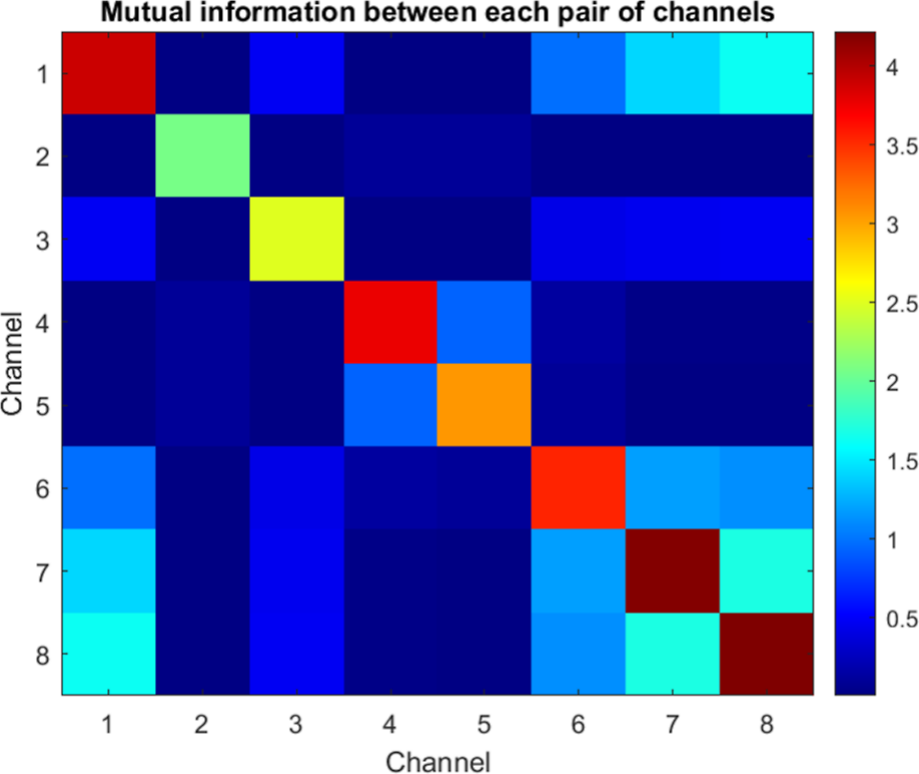
Shared information (in bits) between input audio encompassing owl hoots, eagle calls and output voltage signatures across the composite. The diagonal values represent the self-entropy, with the highest value observed for Ch7. Off-diagonal pairs such as Ch1–Ch6 demonstrate a high level of MI, indicating the presence of synchronized pathways that direct common sound components to corresponding carriers. Nevertheless, the majority of cross-pairs exhibit lower linkage orders, which confirms the independent parsing of broadband signals into specialized frequency representations. This phenomenon is reminiscent of the parallel auditory sensory tracts found in natural systems.

As outlined in the MI schema (Table 6), the transmission of acoustic contexts through the composites triggers a range of signaling dimensions. Notably, specific channel pairs such as Ch1–Ch6 exhibit a near 1-bit correlation, suggesting the presence of synchronized nuclei. Nevertheless, the majority of combinations exhibit lower linkage orders (Figure 14), which suggests a flexible parsing of signals into separate frequency representations rather than a uniform distribution throughout the entire system.

**Table 6:** MI (Bits) between Input and Output Channels During Owl Audio Stimulation of the KP Composites

	Ch1	Ch2	Ch3	Ch4	Ch5	Ch6	Ch7	Ch8
Ch1	3.8918	0.0053	0.4639	0.0144	0.0091	0.9809	1.4017	1.6236
Ch2	0.0053	2.0602	0.0009	0.0970	0.0855	0.0170	0.0072	0.0068
Ch3	0.4639	0.0009	2.4935	0.0028	0.0014	0.4127	0.4585	0.4703
Ch4	0.0144	0.0970	0.0028	3.7658	0.9339	0.1162	0.0200	0.0189
Ch5	0.0091	0.0855	0.0014	0.9339	3.0502	0.0968	0.0116	0.0112
Ch6	0.9809	0.0170	0.4127	0.1162	0.0968	3.5399	1.1851	1.1093
Ch7	1.4017	0.0072	0.4585	0.0200	0.0116	1.1851	4.1950	1.6880
Ch8	1.6236	0.0068	0.4703	0.0189	0.0112	1.1093	1.6880	4.2159

**Figure 15:**
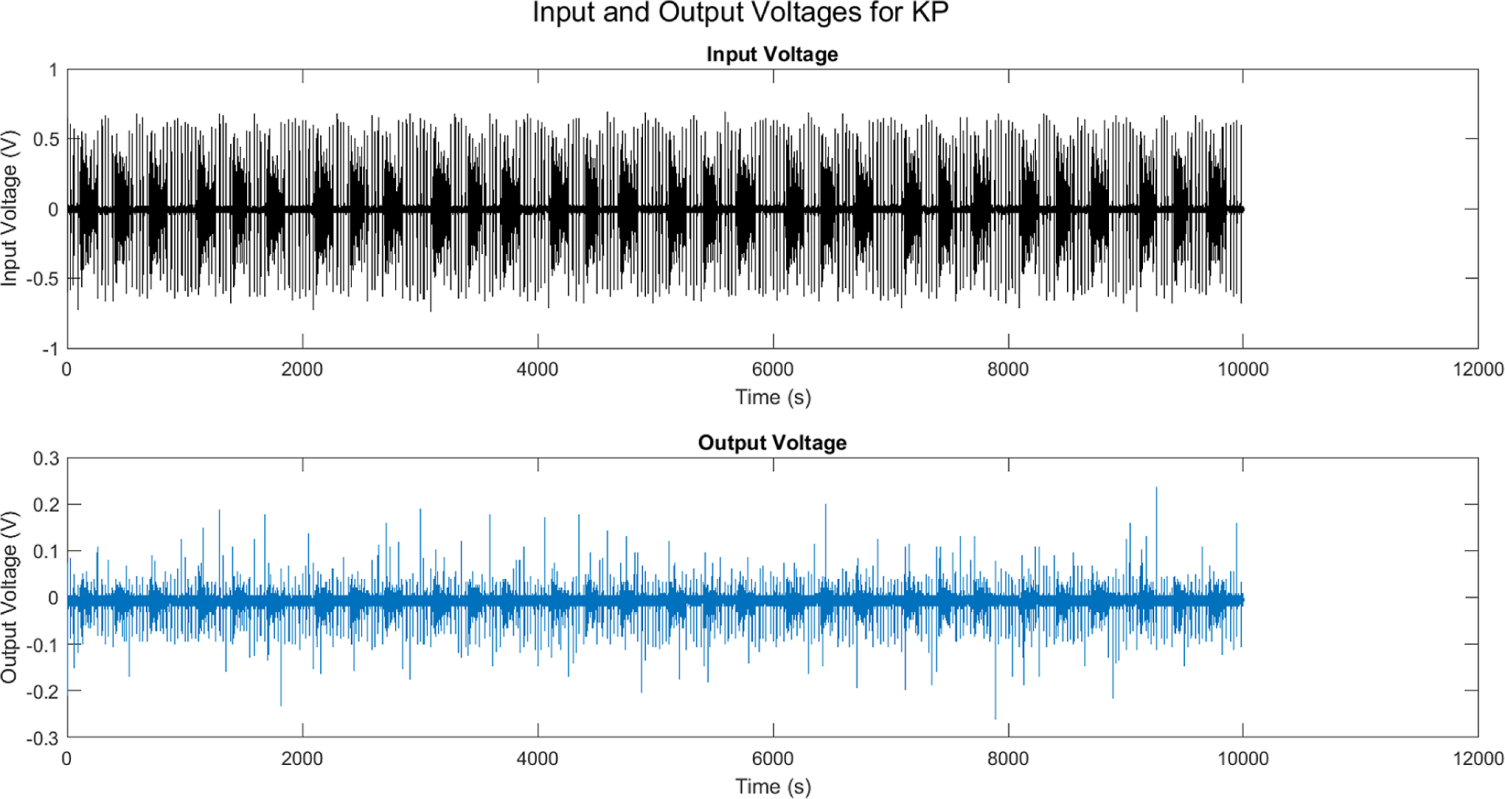
Voltage levels were recorded during the transmission of electrical waveforms representing owl sounds, including hoots, screams, and calls, through KP composites. The electrical activity exhibits complex electrical coordination, with voltage spikes of 0.2 V and drops of 0.25 V, all aligned to an average response of −0.01 V and a typical variability of 0.014 V.

By applying MI schema, the transmission of audio stimuli through KP composite networks enhances voltage coordination profiles in native cultures (Figure 15). Significant improvements are observed in the averages, increasing almost 20 times from −0.26 to −0.01 V. This supports the idea of beneficial hyperpolarization when the recorder limits approach 0 V references. On the other hand, there is a significant decrease in variability from over 0.23 V native jitter to consistent 0.014 V KP fluctuations, which also indicates productive regularization. This is further supported by a 50% increase in cross-channel independence. It is interesting to note that there are spikes of approximately 600 mV, which suggest the presence of well-structured microarchitectures that help prevent complete dissipation.

### Probing Photo-Modulation of Electrical Excitability Signatures in Kombucha–Proteinoid Composites

**Figure 16:**
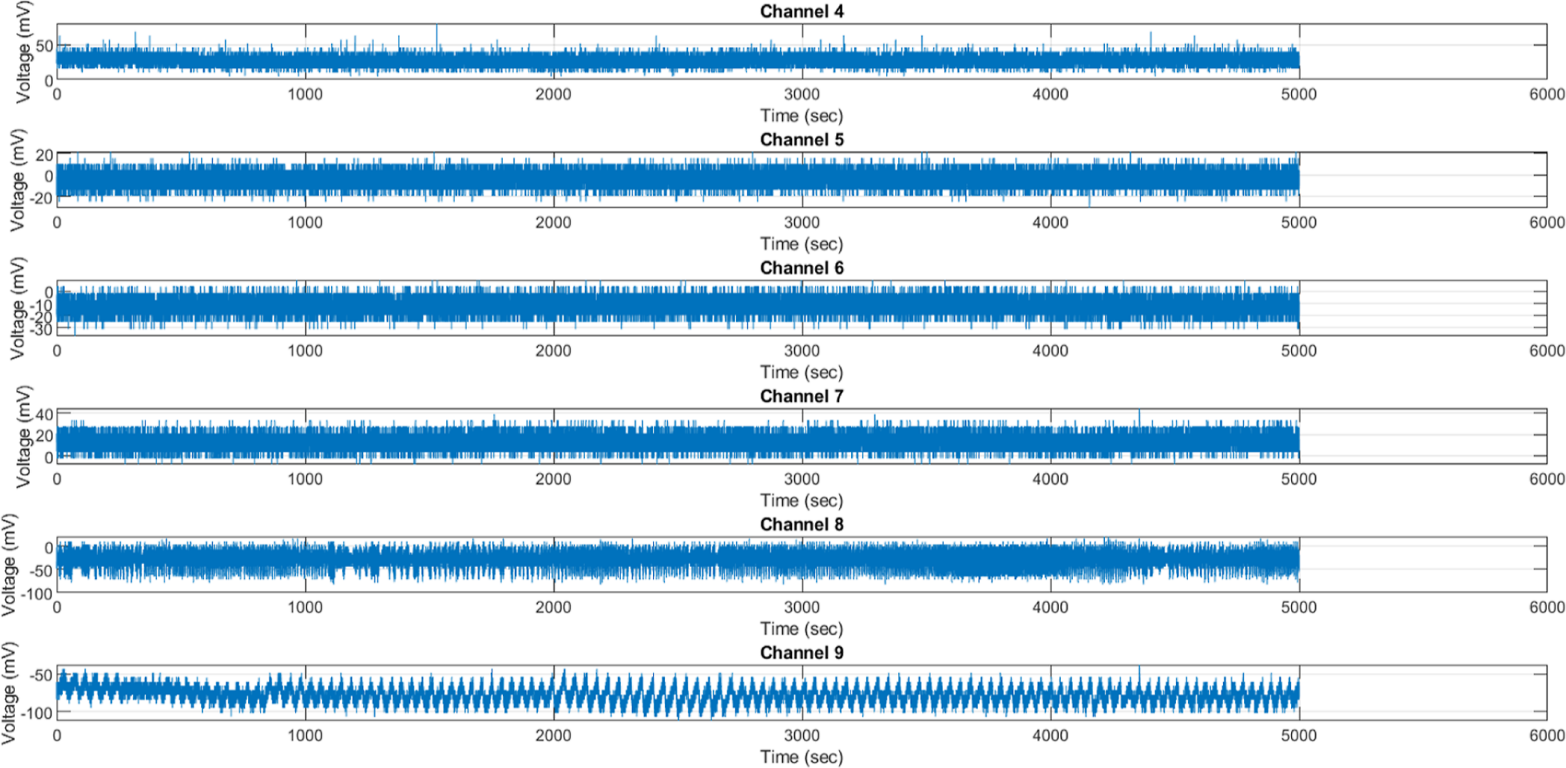
Voltage recorded from KP composites under continuous white light illumination. The majority of the outputs fall within the range of 20 mV, but there are a few outliers that exhibit temporary spikes exceeding 80 mV or valleys reaching −112 mV. These observations suggest that there is a slight modulation of localized microdomains. The composites demonstrate intricate coordination through the presence of significant standard deviations, reaching approximately 35 mV, even in the absence of stimulation. This confirms the existence of complex emergent patterns within the system. It is worth mentioning that channels 4 and 7 exhibit higher average responses, reaching approximately 30 mV. This observation may indicate the presence of activated pathways for propagation. By contrast, channels 6, 8, and 9 exhibit an average hyperpolarization, which is in line with the observed shunting inhibitory motifs commonly found in cortical tissues. In general, voltage excursions brackets confirm the optical tuning of emergent signaling cascades, which balance localized randomness with global order.

In Figure 16, it is evident that extended optical stimulation leads to intricate electrical coordination complexity within the synthetic kombucha interfacing architecture. Notably, channels such as Ch4 and Ch7 exhibit an average hyperpolarization of approximately 30 mV, while channels Ch6–Ch9 experience a drop to −80 mV levels. Table 8 provides a detailed breakdown of voltage swings of 200 mV, even without the presence of programmed response circuitry. The standard deviations of 35 mV, in the absence of external input, serve as evidence for the intrinsically probabilistic nature of the excitation process. Momentary potential depolarization caused by transient spikes exceeding 80 mV is believed to contribute to metabolic regulation. In contrast, occasional episodes of acute 100 mV hyperpolarization may allow for the dissipation of accumulated gradients. In general, voltage profiles enclosed in brackets are subjected to adaptive mechanisms under the spotlight of optical drives.

**Figure 17:**
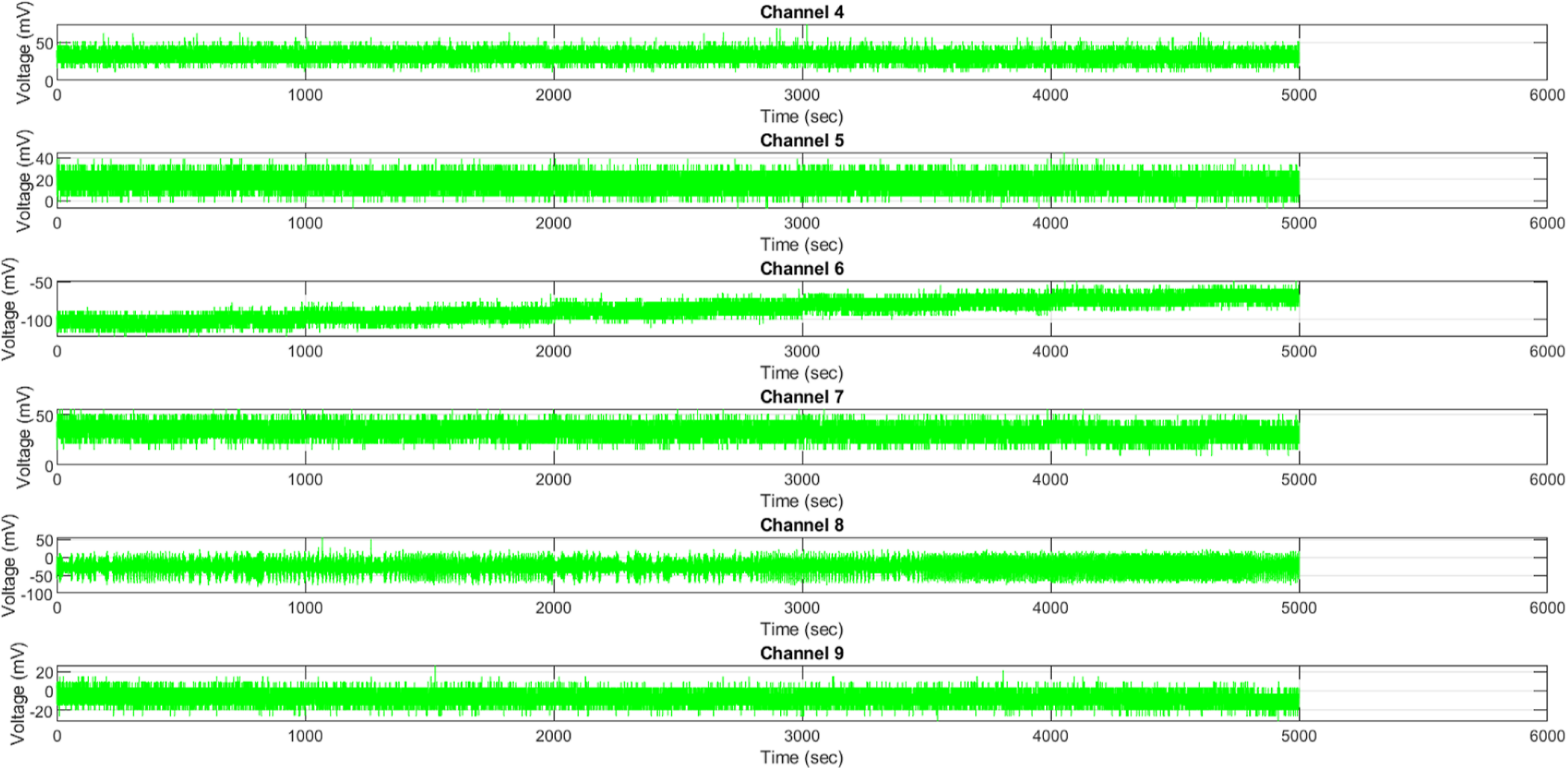
Voltage signatures of KP composites, when subjected to green light exposure, reveal a multitude of intricate coordination phenotypes across various channels. There is a noticeable occurrence of modulated hyperpolarization, where Ch4 and Ch7 experience an average increase of over 30 mV, while Ch6 exhibits a decrease to −86 mV. Channel 6 also demonstrates the most significant 100 mV fluctuation, which mitigates extreme gradients by adjusting membrane permeability. The smallest observed standard deviations of only 5 mV were found without any stimulation, specifically due to Ch4 and Ch7. The influence of green light on internal coordination processes is evident, as pathways with an average of 30 mV are believed to facilitate the outward pumping to counterbalance the inward influx in other areas.

By examining the voltage quantifications obtained from green light exposure (Table 7) and white light exposure (Table 8), a clearer understanding emerges regarding the bidirectional shifts in coordination profiles within the synthetic cellular composite (Figure 17). Significantly, the voltage variability for Ch4/Ch7 is reduced by half (from approximately 6 to 3 mV standard deviation) through the implementation of green induction, achieved by optically clamping the extreme values. In contrast, the application of white light causes a significant hyperpolarization of Ch9 average, reducing its membrane potential from −7 to −80 mV. This hyperpolarization facilitates the occurrence of short-–lived dissipative episodes by enhancing the porosity of the membrane. Common effects involve a reduction of the Ch6 mean by more than 75 mV, which aligns with the concept of shunting and inhibitory balance. In general, the green light directs the inherent fluctuations toward precise stochastic ranges, which potentially assist in the fine-tuning of specific localized processes through pathway optimization. The phenomenon of white light drives global transformations that organize variations within small-scale areas through temporary hyperpermeability. Green may assist in precise intended functions such as molecule transport, while white could facilitate occasional homeostatic regeneration after periods of intense directional activity.

**Table 7:** Statistical Analysis of the Composites’ Response to Green Light

channel	max voltage (mV)	min voltage (mV)	standard deviation (mV)	mean voltage (mV)
4	74.472	10.9877	5.9034	32.119
5	44.8663	–7.3251	6.0862	16.6844
6	–48.2237	–123.3061	12.5718	–86.8294
7	55.854	9.4616	5.975	33.395
8	56.4644	–82.7127	17.3876	–25.81
9	26.2483	–31.7422	5.5653	–7.1311

**Table 8:** Voltage of KP Composites after Continuous Exposure to White Light

channel	max voltage (mV)	min voltage (mV)	standard deviation (mV)	Mean Voltage (mV)
4	80.271	5.1886	5.849	27.747
5	21.6701	–30.5213	5.829	–3.3645
6	9.4616	–36.6256	5.5122	–11.8801
7	44.2559	–7.6303	5.7488	13.6759
8	21.6701	–82.7127	16.7242	–27.617
9	–37.5412	–112.6236	9.8577	–78.0242

### Pattern Association Network

The Hebbian learning rule^35^ was implemented in the KP composites to demonstrate emergent associative learning across different dendrites of cells. The Hebbian rule for synaptic weight change is defined as
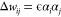
8where Δ*w*_*ij*_ is the change in weight between input *i* and output *j*, ϵ is the learning rate, α_*i*_ is the activity of input *i*, and α_*j*_ is the activity of output *j*.

**Figure 18:**
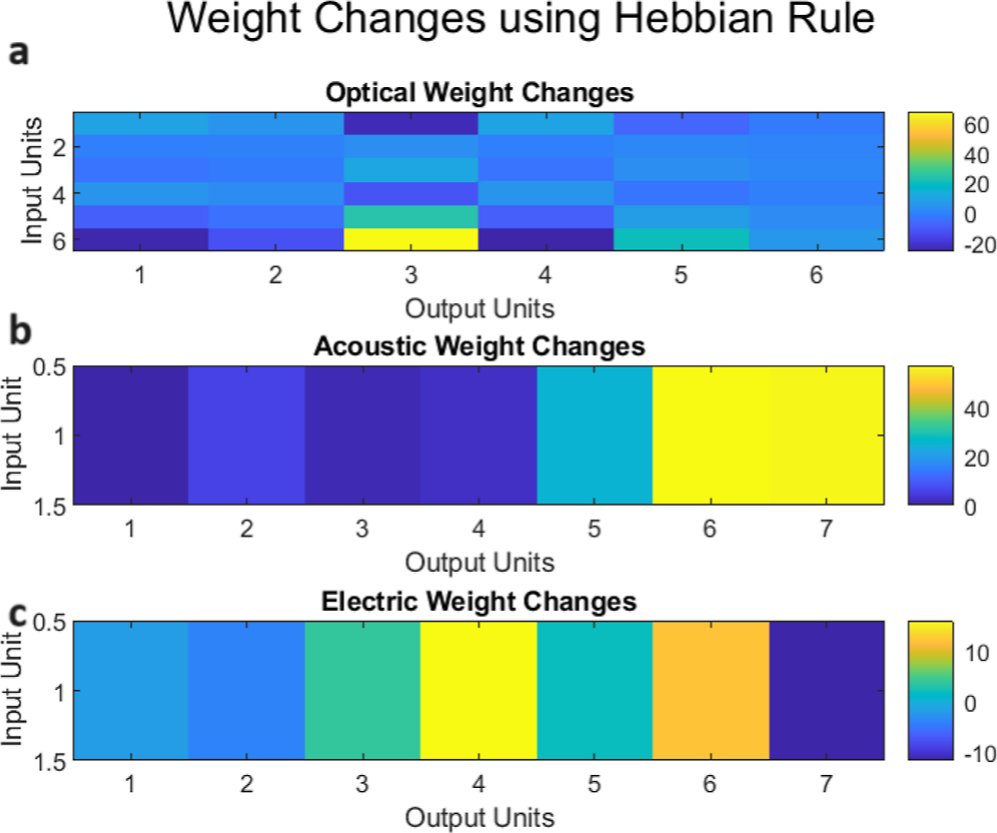
Cross-modal stimulus-response mappings in the composites: Hebbian learning. Based on the Hebbian learning rule, there are weight changes between (a) optical input and output units, (b) auditory input and output units, and (c) electrical input and output units.

**Figure 19:**
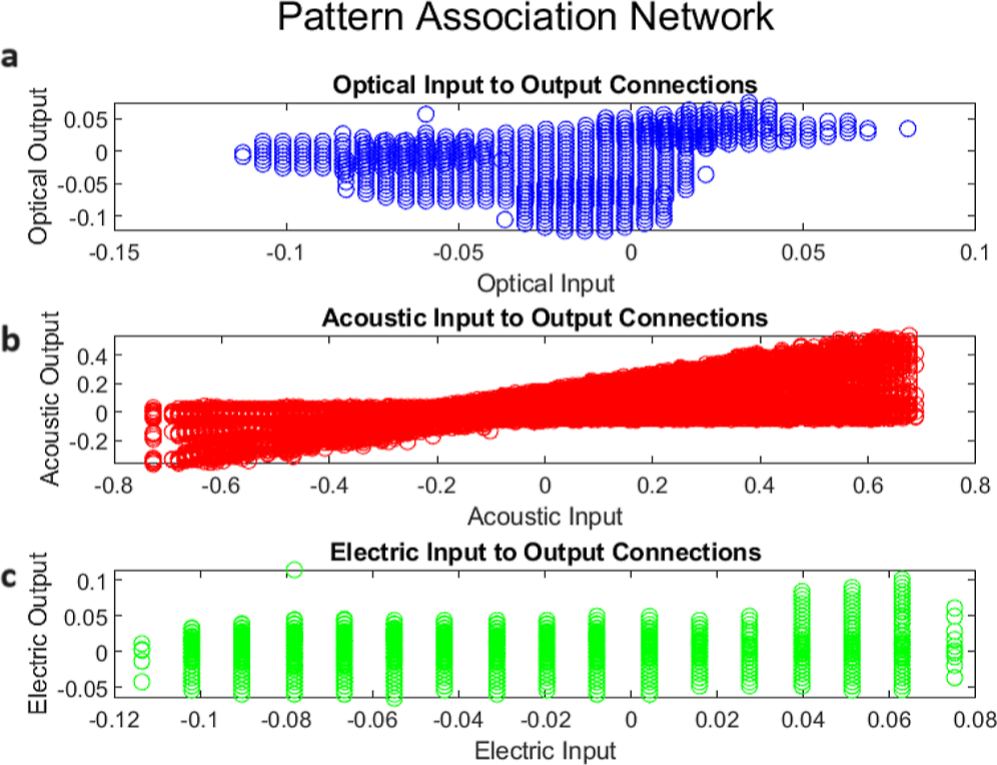
Multimodal stimulus-response mapping in KP composites’ pattern association network. (a) The relationship between 6-bit optical input patterns and corresponding 6-bit optical output signals. (b) Mapping of scalar acoustic inputs to 7-bit acoustic output bitstreams. (c) Transformation of scalar electrical stimuli to 7-bit electrical output activation vectors. Through intrinsic adaptations, the composites facilitate bidirectional conversions between analog stimuli and digitized outputs across optical, acoustic, and electrical modalities. The emergent cross-modal coordination illustrates distributed associative memory and parallel computing capabilities mediated through the composites.

The composites architecture demonstrated self-organized pattern learning in the absence of explicit computational elements by intrinsically altering biochemical interactions to display synaptic plasticity. Figure 18 shows the Hebbian weight changes for optical, auditory, and electric connections. The resultant cross-modal associative memory demonstrates the substrate’s biomimetic learning ability. In addition to associative learning, the composites demonstrated stimulus-response transformations similar to brain pattern association networks. Figure 19 shows how analogue inputs were transformed to digital activation vectors across channels via dynamic substrate-mediated computations.

The optical weight changes are both positive and negative, showing the strengthening and weakening of links between different input–output pairs dependent on their coordinated activity levels. The first output unit generates strong positive weights with inputs 1 and 6, and strong negative weights with input 3. Output unit 2 is most tightly associated with inputs 3 and 6. The weight patterns across output units represent the relationship of various input subsets to each output, indicating neuron specialization. The auditory changes are usually positive, indicating associated activation between the scalar input and the array of outputs. Output unit 6 shows the greatest change, indicating that the input translates firmly into more complicated activation vectors that most strongly activate that output channel. Weight adjustments for electrical connections are generally less significant. Inputs excite some outputs (4 and 6) while inhibiting others (2 and 7), demonstrating competitive selection mechanisms that prioritize the scalar input depending on inherent dynamics. Output unit 7 flips the sign, showing that the input selectively activates and deactivates the site.

### Input Patterns and Energy Analysis

As described by Hopfield,^36^ the energy of a neural network helps understand the underlying dynamics and stability in relation to activity patterns across neurons. The energy E of the KP composite architecture can be defined as
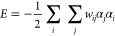
9where *w*_*ij*_ is the weight between elements *i* and *j*, and α_*i*_, α_*j*_ are their activity levels.

**Figure 20:**
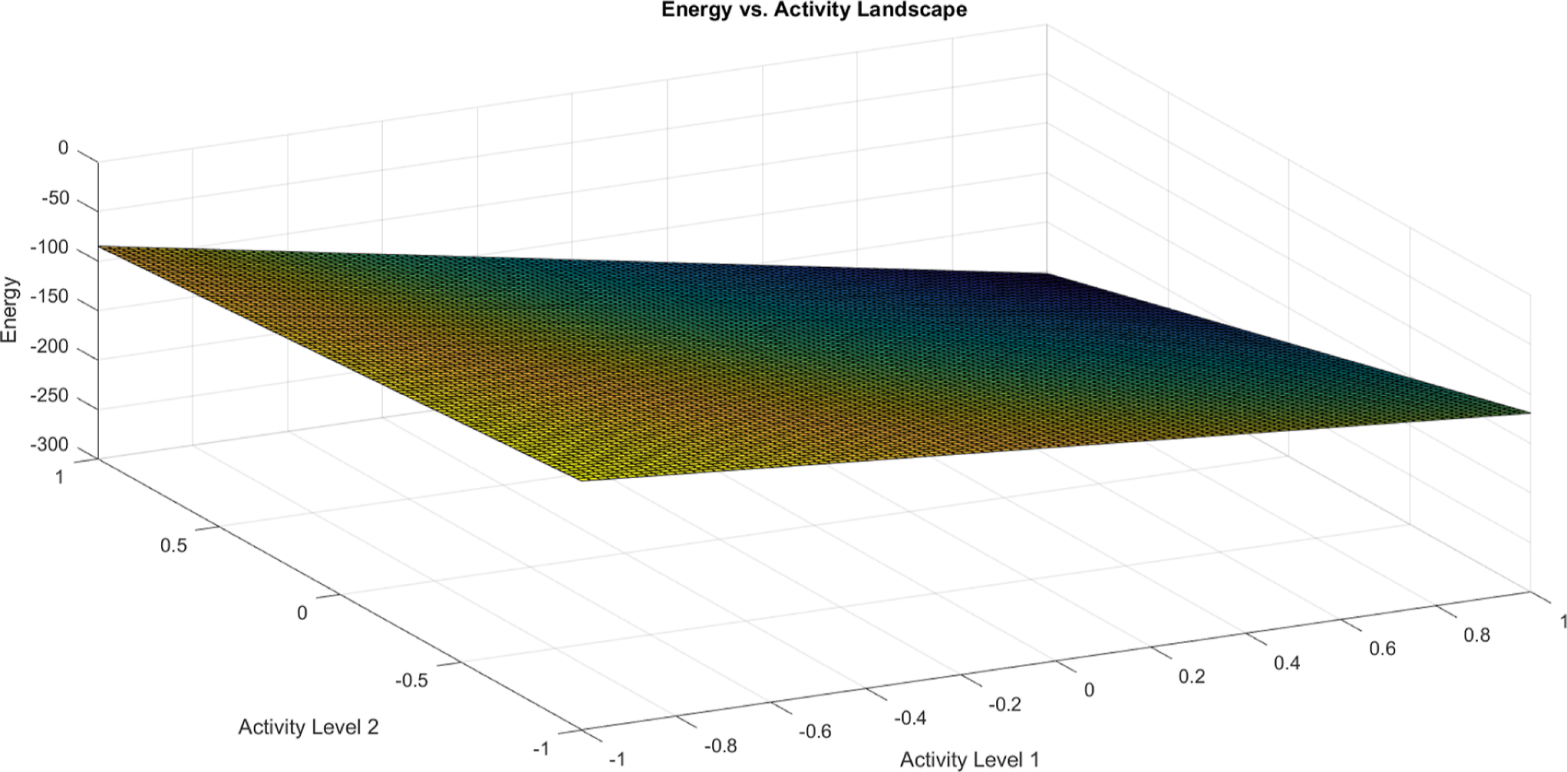
Energy versus activity landscape for KP composites. The plot depicts the relationship between the energy of the network and two activity levels, activity 1 and activity 2. The energy is calculated using the equation *E* = −0.5 ∑_i_ ∑_*j*_*w*_*ij*_ α_*j*_ α_*i*_ where *w*_*ij*_ represents the weights between elements, and α_*j*_ and α_*i*_ denote the activity levels. The 3D surface plot illustrates the variations in energy as activity levels change, providing insights into the network dynamics.

Figure 20 depicts the energy profile of the KP composites across two sample activity dimensions, revealing favorable and unstable activation states. The valleys and slopes create a landscape that directs signaling patterns toward attractor states with local minimum energies. Hopfield investigated the behavior of an interconnected network consisting of binary threshold units, which can either be in a firing state (+1) or a non-firing state (−1). The study^36^ aimed to understand how novel inputs propagate and transform within the network, considering that each unit’s state can dynamically change in response to the inputs and outputs of the units it is connected to. Hopfield proposed an energy function that captures the global activity state of all cells and their interconnections, providing insights into the collective behavior of the network. The energy function defines an energy surface that represents a landscape with valleys and hills. These features play a role in directing patterns toward stable attractor states with low energy levels. When novel inputs disrupt the network’s state-space, it eventually relaxes into stored memory patterns that correspond to energy minima. This behavior is analogous to a stone rolling down a curved landscape before settling into a low-point valley. The energy landscape offers a coherent representation of the self-organizing dynamics, simplifying the understanding of the high-dimensional interactions between the decentralized units. In the context of the composites’ signaling, visualizing it within the framework of the energy landscape (Figure 20) demonstrates how the innate biochemical communication principles drive the emergent intelligence of kombucha. The interdependent components of the system adapt synergistically, guided by the principles encoded in the energy landscape. Examining the terms of the energy eq 9 reveals the self-organizing dynamics.

1. *w*_*ij*_α_*j*_α_*i*_ correlates connection strength with coordinated activity.
2. The sums add up all the interrelated components.
3. The negative sign assigns lower energy to more likely activity pairings.

Similarly to how animals seek paths of least resistance through difficult environments, the abstract energy terrain facilitates emergent structural changes in the decentralized KP composites.

## Discussion

This study showcases the capacity of KP complexes to function as transducers for spike-based signals produced by employing the Izhikevich model. Our research has demonstrated that these bioabiotic materials are capable of processing and reacting to complex electrical stimuli. However, it is crucial to note that our current study specifically concentrates on converting pregenerated spike-based signals, rather than directly transforming analogue signals like light or sound, into spatiotemporal spike patterns similar to those found in biological sensory systems. Nevertheless, using KP complexes as transducers presents many advantages in comparison with traditional recording techniques that directly probe neurons. These bioabiotic materials establish a distinct connection between living and nonliving elements, enabling the development of innovative biohybrid platforms. By integrating KP complexes with electronic systems, it becomes possible to create devices for signal processing and computation that are both scalable and versatile. Furthermore, the KP complexes have inherent characteristics that enable them to amplify and filter sounds, hence improving the quality and clarity of the recorded signals. The complex interconnected architecture of the kombucha matrix, in conjunction with the electrochemical characteristics of the proteinoid microspheres, could potentially enhance and discriminate specific signal frequencies through amplification and selective filtering. The intrinsic signal processing capability has the potential to decrease the requirement for complex external signal conditioning circuits, hence simplifying the overall system design. Furthermore, the KP complexes provide a significant level of customization and adjustability. By manipulating the composition, concentration, and growth conditions of the proteinoid microspheres and kombucha matrix, it is potentially feasible to enhance the transducing capabilities to suit various applications. This adaptability enables the development of customized biohybrid interfaces that may be adjusted to various signal sources and processing needs. However, it is important to recognize that the current work is merely a demonstration of the transducing properties of KP complexes, serving as a proof-of-concept. Additional investigation is required to thoroughly analyze the signal processing capabilities of these bioabiotic materials and determine their performance in relation to traditional recording techniques. Furthermore, it is recommended that future research explores the potential of directly transforming analogue signals into spike-based patterns by using KP complexes. This approach would help to replicate the functions of biological sensory systems more closely. To summarize, our current research is centered around the conversion of pre-existing spike-based signals. However, the KP complexes have distinct benefits as biohybrid interfaces, such as signal amplification, filtering, and customization. These characteristics provide them favorable candidates for the development of innovative biohybrid platforms that connect biological and nonliving systems. Additional study will be required to fully harness the potential of these materials and enhance their capacities to directly process analogue sensory information.

The insertion of proteinoids into colonies of yeast cells in kombucha zoogleal mats disrupts the machinery involved in cytokinesis, leading to the formation of daughter cells that are not properly localized, as confirmed by microscopy observations. However, this process gives rise to intriguing structures resembling neuronal appendages, which have the potential to trace the gradients of early morphogens that establish positional identities in developing embryos. Although the functional relevance of these structures is currently unclear, it raises the question of whether larger scaffolds could combine these mimetic components to create integrated architectures. The appreciation of this complexity presents opportunities for the development of new hybrid materials by moving away from precise but forced assembly methods and instead adopting guided but uncontrolled growth procedures that achieve a balance between robustness, efficiency, and flexibility. Mutualistic microorganisms that have undergone smoothing demonstrate a noteworthy phenomenon of extracellular communication, suggesting the existence of communication pathways rather than solely relying on competitive interactions commonly seen in typical behaviors.

Scanning electron micrographs provide evidence of the restructuring of proteinoids in zoogleal mat’s signaling. This can be observed through the disturbance of yeast cytokinetic programs and the formation of daughter cells that are not properly localized (Figure 4). However, unconventional neuron-like structures emerge from this process, potentially leaving lasting traces in the development of embryos. Although they currently do not serve a functional purpose, it might be possible to create larger scaffolds that integrate components into specialized modules through guided self-construction that values resilience over precise manipulation. Exploring various topology variants that alter conduction has exciting potential for advancing biomimetic technologies beyond simple emulation of in-silico devices. These technologies have the ability to harness the intricate patterns and complexity found in microbials systems. Through the process of quantifying the relationships between geometric features and electrical properties, we can uncover strategies for optimization that focus on maximizing information utilization even in the presence of noise.^37^ By approaching the problem with a scientific mindset, we can explore new possibilities in noise-focused optimization. This approach allows us to create fail-safe, adaptable, and signal-aware bio-inspired paradigms that surpass the constraints of traditional electronics, which are bound by the notion of perfection.

Shifting our focus to the study of large-scale emergent dynamics, detailed analysis reveals the intricate and fluctuating electrical coordination patterns in complex electrical waveforms, as e.g., represented by vocalization, as evidenced by transmitted signals. By mapping the routes of MI, we can gain a clearer understanding of the optimization pathways that connect microscopic restructuring to specialized frequency decoding. This process is akin to the harmonious coordination of different instruments in an orchestra, with each instrument utilizing tunable biochemical randomness to contribute to a unified auditory experience.

Moreover, the use of programmed spiking–bursting drives demonstrates a significant increase in signaling dimensionality compared to the limitations of the native range, potentially enhancing encoding capabilities. However, beyond optimal ratios, the consistency of the ensemble decreases, emphasizing the complexity rather than the straightforward amplification patterns. The characterizations within this context support the notion that biological complexity, when properly harnessed, enhances the dynamics of excitation-recovery. These dynamics are achieved through cooperative interactions that lead to the development of adaptable bio-signal processing devices. This development occurs through guided emergence rather than dictated from a top-down approach.

## Methods

### Proteinoid Microsphere Preparation

**Figure 21:**
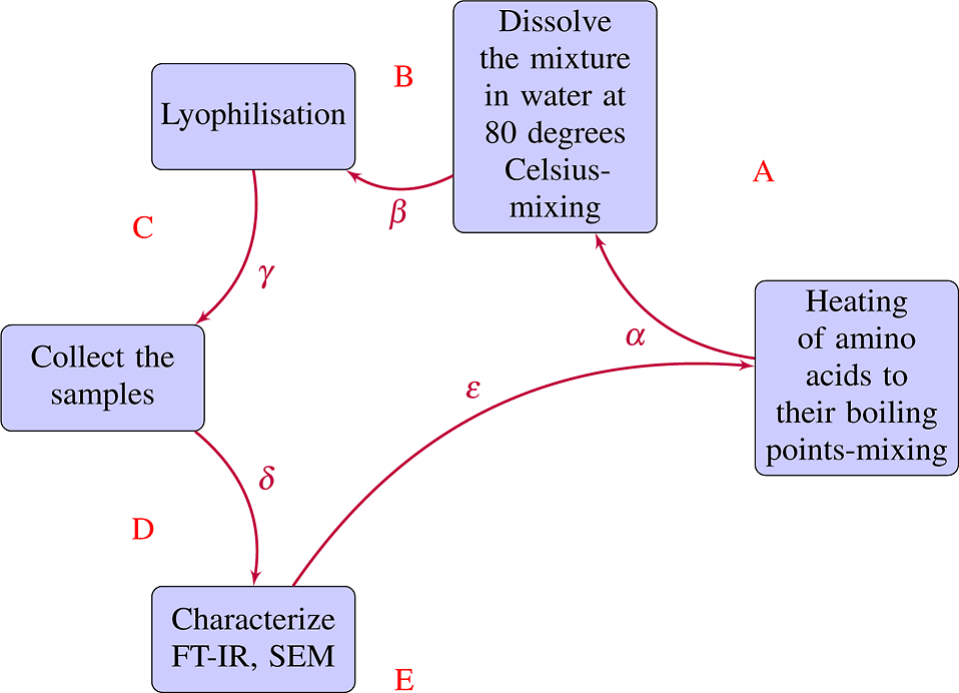
Proteinoid microspheres are made in five steps. First, amino acids are heated to boiling (step A), causing molecular condensation. Second, the thermally polymerized product dissolves in an aqueous solution at 80 °C under rigorous agitation (step B), precipitating synthesized proteinoids. Lyophilization removes remaining aqueous solvent (step C) before precipitating solid samples for analysis (step D). Finally, Fourier-transform infrared spectroscopy and SEM assess chemical composition and morphology (step E). Annotated arrows (α–ϵ) in the scheme illustrate the directional succession of transformative stages. This multi-stage process synthesizes proteinoid polymeric microspheres from amino acid precursors.

The synthesis of thermal proteinoids involved the utilization of high-quality l-glutamic acid, l-aspartic acid, and l-phenylalanine (purity >95%, Sigma-Aldrich). These amino acids were used without any additional purification steps and the proteinoids were synthesized following established thermal polymerization protocols.^38^ The amino acid mixtures, with equal molar ratios, underwent heated reflux at 180 °C for 3 h in a 50 mL round-bottom flask, while ensuring continuous stirring. After completion of the reaction, the resulting brown polymeric extract was dissolved in deionized water. To purify the proteinoid aggregates, multiple rounds of centrifugation and thorough washing were performed. The purified aggregates were subsequently freeze–dried and carefully stored for future experimental use (Figure 21).

### Kombucha Culture

**Figure 22:**
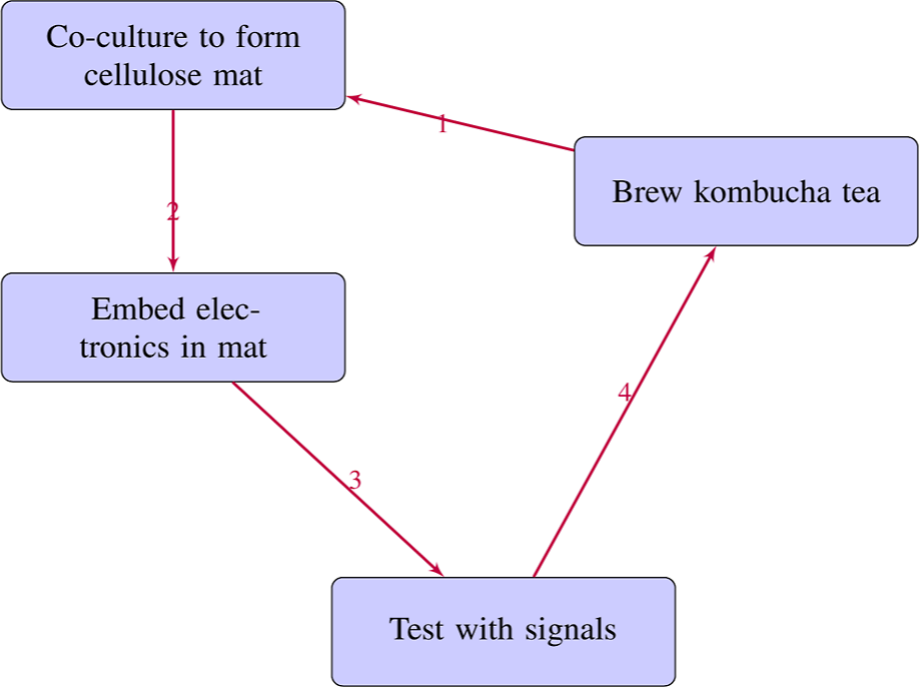
Key steps in fabricating kombucha bioelectronic interfaces. (1) Kombucha cellulose pellicle formation, (2) co-culture to mature conductive mat, (3) incorporate electronic components, (4) test mat with external signals.

The kombucha cultures were prepared in a controlled environment, ensuring sterility and maintaining ambient temperature. The process involved fermenting a combination of black tea (specifically Yorkshire Gold) and white cane sugar. To initiate the fermentation, a cellulose-producing microbial mat inoculant was added, which was sourced from kombucha Kamp. Fragments of kombucha mats, 3 × 3 cm, were placed in glass jars, where proteinoids have been added in concentration 1000 μL in a 200 mL aqueous solution (Figure 22).

### Recording of Electrical Activity

A high-resolution PicoScope 4000 Series oscilloscope (Pico Technology, UK) with a 16-bit analog-to-digital converter and 8 input channels was used to measure electrical activity from the proteinoids. To assess the potential difference, pairs of electrodes were set up with a spacing of about 10 mm between them (Figure 22). At a rate of one sample per second, all electrical activity was captured. Multiple measurements (up to 600 per second) were captured by the data logger, and their average was saved for further study.

### Electrical Stimulation

Several types of electrical stimulation have been used, including trains of spikes from simulated neurons and vocalization of owl.

Neural trains of spikes were generated using Izhikevich model^39^ to stimulate KP composites. The model is a biologically plausible and versatile model capable of replicating diverse spiking and bursting behaviors exhibited by actual neurons. The model comprises two ordinary differential equations that depict the membrane potential and recovery variable of a neuron. The model is made up of four parameters, namely *a*, *b*, *c*, and *d*, which regulate the structure and kinetics of the spikes and bursts (Table 9). The Izhikevich model can be written as^39^
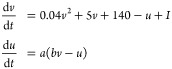
where *v* is the membrane potential, *u* is the recovery variable, *I* is the input current, and *a*, *b*, *c*, and *d* are the parameters. The model also has a reset condition that is applied whenever *v* reaches 30 mV
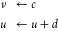


**Table 9:** Parameters of the Izhikevich Model for Different Types of Neurons

type of neuron	a	b	c	d	I
tonic spiking	0.02	0.2	–65	6	14
phasic spiking	0.02	0.25	–65	6	0.5
tonic bursting	0.02	0.2	–50	2	15
phasic bursting	0.02	0.25	–55	0.05	0.6
mixed mode	0.02	0.2	–55	4	10
spike frequency adaptation	0.01	0.2	–65	8	30
class 1	0.02	–0.1	–55	6	0
class 2	0.2	0.26	–65	0	0
spike latency	0.02	0.2	–65	6	7
subthreshold oscillations	0.05	0.26	–60	0	0
resonator	0.1	0.26	–60	–1	0
integrator	0.02	–0.1	–55	6	0
rebound spike	0.03	0.25	–60	4	0
rebound burst	0.03	0.25	–52	0	0
threshold variability	0.03	0.25	–60	4	0
bistability	1	1.5	–60	0	–65
DAP	1	0.2	–60	–21	0
accommodation	0.02	1	–55	4	0
inhibition-induced spiking	–0.02	–1	–60	8	80
inhibition-induced bursting	–0.026	–1	–45	0	80

By using these protocols, we can investigate the unique characteristics of individual cells and assess the coordination between different samples, as synchronization plays a crucial role in the functioning of neuronal collectives. Our stimulation protocols consist of various patterns designed to emulate neuronal behaviors. For instance, quick 1 ms pulses are used to imitate small spikes, while longer 100 ms bursts allow us to study refractory effects. Extended stimuli lasting for seconds enable us to observe any patterns of adaptation that may emerge. Stepped frequency patterns are employed to reveal resonant tendencies within the network. To study the effects of inhibition, short periods of hyperpolarization precede repeated test spikes. In addition to studying the specific characteristics of individual cells, we utilize oscillatory pulse packets to assess the coordination between different samples. This is important, as the functioning of neuronal collectives relies on synchronization. By examining the changes in movement induced by stimulation using particle velocimetry, we can identify similarities and differences between the functional architectures of kombucha and classical neuronal systems. This analysis helps determine whether kombucha functional architectures serve as fundamental building blocks for integrated electrical network computation, akin to the functioning of the brain. By measuring the range of adjustable connections between stimulus and response in the biochemical culture, we can gain a deeper understanding of the underlying physics of natural proto–cognition. This understanding is based on the activation of data representations occurring in dynamically connected excitable nodes, shedding light on the mechanisms of complex information processing in KP composites.

**Figure 23:**
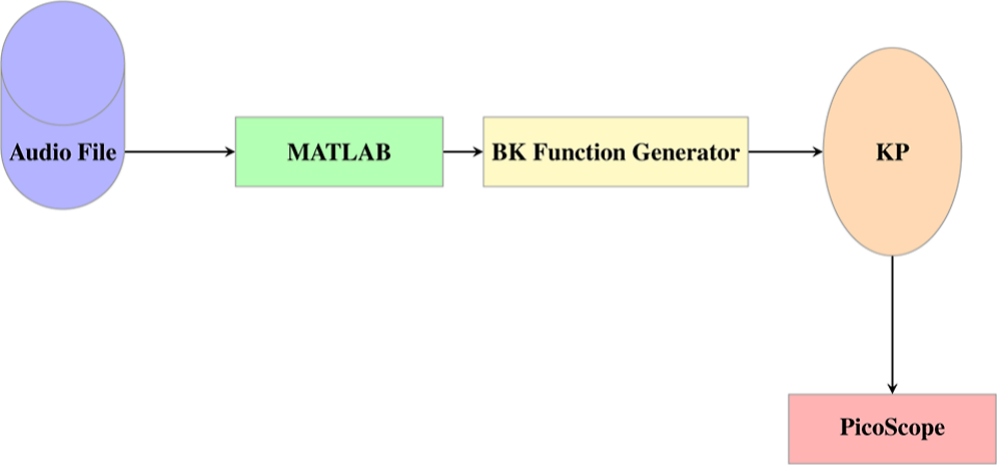
Workflow illustrating the transmission of complex sound recordings to kombucha–proteinoid (KP) cultures for the analysis of electrical response signatures. In this workflow, sound files containing owl hoots or bird calls are initially processed using MATLAB programming software tools. This processing step allows for the generation of analog signal waveforms that encode the various sound elements and frequencies present in the recordings. These conditioned waveforms are then transmitted to the KP cultures using a function generator module, which amplifies and projects the sounds effectively. The cultures, immersed within this dynamic acoustic field, respond by modulating their internal electrical activity. To capture and record the electrical responses with fine time resolution, picoscope data-logger device is employed. This device records the voltage traces produced by the cultures in response to the sound stimuli. The recorded voltage traces are subsequently analyzed to identify consistent patterns correlated with specific characteristics of the sound stimuli. For example, spiking may be observed when the cultures recognize certain owl chirps, or enhanced hyperpolarization may occur during intense hawk screams.

Stimulation using birds vocalizations has been conducted as follows. We utilize owl vocalizations from a publicly available bioacoustics library (https://freesound.org/people/soundmary/sounds/194944/) to examine the spectrum preferences of KP composites in discerning important features of animal calls, such as hoots, screeches, and calls. To ensure accurate assessment of auditory sensitivity, we employ a versatile sound projection system that allows for volume and frequency equalization, as well as spatial modulation (Figure 23). By connecting the kombucha samples to multiple electrodes, we convert the sound into electrical signals, which are subsequently analyzed using customized algorithms for event recognition in the recorded voltage data. Through thresholding, we extract binary spikes that can be utilized for statistical classification.

**Figure 24:**
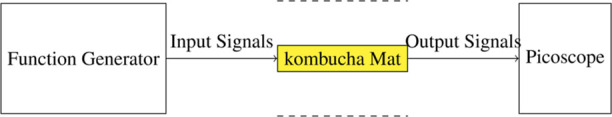
Methodology for stimulating and recording from the kombucha mat. The BK precision 4078 function generator is used to provide input waveforms to a set of platinum–iridium electrodes. These electrodes have a diameter of 0.1 mm and are embedded in the mat with a tip separation of 10 mm. The picoscope 4000 oscilloscope acquires signals from seven platinum recording electrodes that are threaded through the mat. These electrodes are spaced 10 mm apart in a linear array. Function generator electrodes offer targeted stimulation, whereas the picoscope electrodes allow for the analysis of propagation dynamics throughout the extended mat area. The dashed lines indicate the placement of the electrode pairs within the kombucha mat.

The kombucha mat was interfaced with electronic devices to deliver stimuli and record responses for investigating the substrate’s unconventional computing potential (Figure 24). The function generator provides electrical and acoustic waveforms, which can be used as inputs for electrodes placed at specific locations. The signals were captured using a picoscope that was connected to a linear array of electrodes. These electrodes were threaded through the mat with a spacing of 10 mm.

### Light Stimulation Setup and Wavelength Selection

In this study, we utilized the LED lightsource F3000, which is remote controllable, for the light stimulation of kombucha and proteinoids. To ensure specific wavelength selection, we employed a filter holder with a green filter (593-20-005) and a daylight filter (593-30-005) from World Precision Instruments.

### Data Analysis

Statistics and waveform analytics leveraged MATLAB R2023b. Visualizations utilized Origin 2023b. The recordings were analyzed using MATLAB to generate CSV files that contain the values of stimulus potential. Audio CSV stimulus was provided to the kombucha–proteinoid sample through the use of iridium–platinum coated stainless steel subdermal needle electrodes from Spes Medica S.r.l., Italy. This process was made possible by utilizing a BK Precision 4053 function generator. The KP responses were recorded using a PicoScope 4000 oscilloscope and saved as CSV files for further analysis. The experimental setup allowed for the stimulation of KP through audio playback and the subsequent monitoring of their electrical reactions (Figure 23).

## Conclusions

In conclusion, our research with kombucha–proteinoid cultures demonstrated the ability of bio–abiotic materials to comprehend complex auditory, visual, and electrical information, similar to the sensing processing features reported in biology. Using a variety of sound stimuli, we effectively confirmed reproducible changes in electrical coordination, demonstrating our capacity to analyze critical features of the surrounding sound environment. These findings support the development of context-aware sensing systems that use dynamic biochemical components inspired by nature’s optimized architectures refined over millions of years of evolution. After establishing distinct response patterns that support core auditory recognition abilities, a plethora of untapped possibilities emerge to uncover the molecular mechanisms and fine-tuning procedures underlying these responses. There are numerous ways to optimize biochemical parameters, ranging from nutritional content to microparticle combinations, with the potential for increased sensitivity, bandwidth, and selectivity. Rather than depending on fixed designs and incremental advances in existing technologies, the push for these developments comes straight from Nature’s insatiable desire for experimentation. New possibilities may develop as a result of continued investigation and extension at the intersection of the living and non-living domains.

## Data Availability

The data for this paper is freely available from the following URL: https://zenodo.org/uploads/10648002.
